# Genome-wide identification and analysis of a cotton secretome reveals its role in resistance against *Verticillium dahliae*

**DOI:** 10.1186/s12915-023-01650-x

**Published:** 2023-08-04

**Authors:** Ran Li, Xi-Yue Ma, Ye-Jing Zhang, Yong-Jun Zhang, He Zhu, Sheng-Nan Shao, Dan-Dan Zhang, Steven J. Klosterman, Xiao-Feng Dai, Krishna V. Subbarao, Jie-Yin Chen

**Affiliations:** 1 State Key Laboratory for Biology of Plant Diseases and Insect Pests, Institute of Plant Protection, Chinese Academy of Agricultural Sciences, Beijing, 100193 China; 2 Western Agricultural Research Center, Chinese Academy of Agricultural Sciences, Changji, 831100 China; 3 The Cotton Research Center of Liaoning Academy of Agricultural Sciences, National Cotton Industry Technology System Liaohe Comprehensive Experimental Station, Liaoning Provincial Institute of Economic Crops, Liaoyang, 111000 China; 4 United States Department of Agriculture, Agricultural Research Service, Salinas, CA USA; 5 Department of Plant Pathology, University of California, Davis c/o United States Agricultural Research Station, Salinas, CA USA

**Keywords:** Cotton, Verticillium wilt resistance, Secretome, Defense response

## Abstract

**Background:**

The extracellular space between the cell wall and plasma membrane is a battlefield in plant-pathogen interactions. Within this space, the pathogen employs its secretome to attack the host in a variety of ways, including immunity manipulation. However, the role of the plant secretome is rarely studied for its role in disease resistance.

**Results:**

Here, we examined the secretome of Verticillium wilt-resistant *Gossypium hirsutum* cultivar Zhongzhimian No.2 (ZZM2, encoding 95,327 predicted coding sequences) to determine its role in disease resistance against the wilt causal agent, *Verticillium dahliae*. Bioinformatics-driven analyses showed that the ZZM2 genome encodes 2085 secreted proteins and that these display disequilibrium in their distribution among the chromosomes. The cotton secretome displayed differences in the abundance of certain amino acid residues as compared to the remaining encoded proteins due to the localization of these putative proteins in the extracellular space. The secretome analysis revealed conservation for an allotetraploid genome, which nevertheless exhibited variation among orthologs and comparable unique genes between the two sub-genomes. Secretome annotation strongly suggested its involvement in extracellular stress responses (hydrolase activity, oxidoreductase activity, and extracellular region, etc.), thus contributing to resistance against the *V. dahliae* infection. Furthermore, the defense response genes (immunity marker *NbHIN1*, salicylic acid marker *NbPR1*, and jasmonic acid marker *NbLOX4*) were activated to varying degrees when *Nicotina benthamiana* leaves were agro-infiltrated with 28 randomly selected members, suggesting that the secretome plays an important role in the immunity response. Finally, gene silencing assays of 11 members from 13 selected candidates in ZZM2 displayed higher susceptibility to *V. dahliae*, suggesting that the secretome members confer the Verticillium wilt resistance in cotton.

**Conclusions:**

Our data demonstrate that the cotton secretome plays an important role in Verticillium wilt resistance, facilitating the development of the resistance gene markers and increasing the understanding of the mechanisms regulating disease resistance.

**Supplementary Information:**

The online version contains supplementary material available at 10.1186/s12915-023-01650-x.

## Background

Plants are confronted by a variety of pathogens, and they rely on their defense network to resist infection since they are immobile [1]. During the plant-pathogen interactions, the extracellular space between the cell wall and the plasma membrane acts as the initial battlefield [2], where there occurs a “joust” for life or death. Thus, both host plants and pathogens employ diverse evolutionarily honed strategies to knock out their opponent.

Pathogens employ their respective secretomes to gain advantage during host infections. The secretome, comprising multiple pathogenic factors, plays diverse functions in the infection process [3], including cell wall degradation, scavenging host reactive oxygen species, suppressing host immunity, and acquisition of nutrition [4]. For example, hydrolases in the secretome are considered important for the generation of disease symptoms and pathogenesis, especially those involved in plant cell wall degradation, important for destroying physical barriers in the plant [5]. Thus, many pathogens have an expanded arsenal of the carbohydrate-active enzymes (CAZymes) to degrade plant cell walls (especially pectin and cellulose) and to promote successful infection and colonization of their hosts [6–8]. Furthermore, pathogens secrete hundreds of effectors that shield the pathogens from the host’s immune responses or from the manipulation of host cell physiology [9, 10]. For instance, the hemibiotrophic fungal pathogen *Verticillium dahliae* secretes effectors that suppress plant defense responses for successful infection, including the cellulose-binding protein VdCBM1 [11], isochorismatase VdIsc1 [12], and small cysteine-rich protein VdSCP41 [13]. Overall, the pathogen secretome plays a crucial role on the front lines of the battlefield between the pathogen and its host.

Conversely, host plants also have evolved strategies to activate defense responses for restricting pathogen proliferation [14]. Plants employ two classical immunity networks in response to pathogen attacks [15, 16]. The first defense system is a basal defense activated by conserved pathogen-associated molecular patterns (PAMPs) that are recognized by plant cells via pattern recognition receptors (PRRs) [17]. This defense has been termed PAMP-triggered immunity (PTI) and involves the rapid activation of downstream defense responses [17], which stimulate a second immune system known as effector-triggered immunity (ETI). After breaching the first defense system, ETI involves additional resistance proteins (R) that recognize specific pathogen effectors, resulting in the rapid activation of the defense responses [15, 16]. The plant secretome plays a critical role against pathogens, which involves the maintenance of cell wall structure, sensory functions between the host and the pathogen, communication between plant cells, etc. [18]. Extracellular vesicles (EVs, lipid bilayer-enclosed, cytosol-containing spheres) released into the extracellular environment play important roles in disease resistance by physically preventing penetration, inhibiting pathogen proliferation by transmitting toxic molecules, and regulating immune signaling in the form of removing molecular regulators from the cell surface [19, 20].

More specifically, the plant secrotome provides a multipronged protection against reactive oxygen species (ROS, oxalate oxidases, superoxide dismutases, peroxidases, singlet oxygen, etc.), antifungal activity (pathogenicity-relate protein 1 (PR1), lipases, proteases, lectines, chitinases, glucanases, etc.), cell wall remodeling (polygalacturonases, xylanases, etc.), and activation of immune response through the perception of cell wall degradation products generated by the plant secretome (chitinases, glucanases, polygalacturonases, etc.) [2, 21, 22]. For instance, the members of the pathogenesis-related protein 1 (PR1) family are among the most abundantly secreted protein in plants during pathogen infection, which is activated by salicylic acid signaling [23]. Plant-derived proteases are enriched in the apoplastic region during host–pathogen interactions, where they act to enhance host resistance against different types of pathogens [24–26]. Subtilases (SBTs) belonging to the serine protease family are involved in pathogen resistance in plants [27, 28] and enhance mitogen-activated protein kinase, defense gene expression, and resistance against bacterial and fungal pathogens [27]. The secreted aspartic protease (TiAP1) of *Thinopyrum intermedium* interacts with the *Blumeria graminis* f. sp. *tritici* chitin deacetylase (BgtCDA1), inactivating its deacetylation function, rendering fungal cell walls susceptible to the wheat-secreted chitinases that liberate chitin fragments and further activating host immune responses [29]. Therefore, the plant secretome acts at the front line of defense and plays pivotal roles in disease resistance against pathogens.

Cotton is an important crop worldwide because of its fiber and oil seeds, and Verticillium wilt caused by *V. dahliae* is the most destructive disease of cotton, reducing yield and fiber quality on over 50% of cotton acreage [30]. Verticillium wilt is difficult to control due to the broad host range of *V. dahliae*, its long-term survival in soil, and its niche in the plant vascular system which is not amenable to fungicides [31]. For these reasons, improving genetic resistance is considered the optimal method to manage Verticillium wilt [32]. Thus, the identification of resistance genes in cotton has been a priority by using the Verticillium wilt-resistant germplasm from *Gossypium barbadense* since the commonly cultivated *Gossypium hirsutum* lacks complete resistance against *V. dahliae* [33, 34]. Within *G. barbadense*, a number of genes involved in Verticillium wilt resistance have been identified, including *G. barbadense* NB-ARC domain-containing 1 (GbaNA1) [30], nucleoredoxin 1 (GbNRX1) [35], cinnamyl alcohol dehydrogenase GbCAD1 and suppressor of SA insensitive 2 (GbSSI2) [36], subtilase 1 (GbSBT1) [37], Ser/Thr protein kinase (GbSTK) [38], and cysteine-rich receptor-like kinase GbCRK18 [39]. Several genes from other cotton species were also identified for their roles in Verticillium wilt resistance, including the *G. hirsutum* polyamine oxidase (GhPAO) [40], villin 4 (GhVLN4) [41], polygalacturonase-inhibiting protein 1 (GhPGIP1) [42], and *G. hirsutum* dominant suppressor of camta3 1 (GhDSC1) [43]. These resistance genes activate diverse defense responses, including the regulation of hormone levels, enhancing the scavenging of reactive oxygen species, activating the expression of the pathogenesis-related genes, and accelerating phytoalexin (gossypol) synthesis [44, 45]. For instance, the silenced *GhWAKL* compromised Verticillium wilt resistance in cotton, which mainly inhibited the defense response by suppressing salicylic acid signaling [45, 46]. However, only a few secreted proteins have been reported to function in cotton Verticillium wilt resistance, such as chitinase [47, 48]. Thus, additional roles of secretome in Verticillium wilt resistance remain unknown.

The availability of higher-quality genome sequences of *G. hirsutum* cultivar Zhongzhimian No.2 [49] has enabled bioinformatics studies to identify candidate genes encoding secreted proteins that hold promise in the development of resistance in *G. hirsutum*. Cultivar Zhongzhimian No.2 (ZZM2) is the most widely planted Verticillium wilt-resistant cultivar in China [50–52], covering over 7.9 million ha (reported by the Chinese Ministry of Agriculture and Rural Affairs [MARA] in 2021). We previously sequenced the whole genome of ZZM2, revealing a genome size of 2.33 Gb, encoding 95,327 predicted coding sequences [49]. The main objectives of this study were (i) to identify the secretome among predicted coding sequences from cv. Zhongzhimian No.2 genome, (ii) to elucidate the sequence and functional clustering characteristics of this secretome, (iii) to identify the predicted defense response functions of the secretome during *V. dahliae* infection by transcriptome analyses, and (iv) to functionally analyze components of the plant secretome that have predicted roles in Verticillium wilt resistance in cotton.

## Results

### Identification of the cotton secretome from bioinformatic analyses

Secreted proteins of the cotton secretome were identified in silico based on the presence of a signal peptide, a lack of a transmembrane domain, and predicted extracellular location [8]. In this study, the 2.33-Gb genome sequence of Verticillium wilt-resistant upland cotton, ZZM2 [49] (DDBJ/ENA/GenBank accession is JAMQUR000000000; BioProject accession is PRJNA846595), was employed for the prediction of the secretome. Among the 95,327 predicted coding sequences, 8383 proteins were identified with a signal peptide sequence using SignalP 5.0 [53]. An overlapping 3879 proteins had characteristics of extracellular localization as predicted with the plant model in WolfPsort [54]. In total, 74,991 and 76,025 proteins were identified without transmembrane (TM) motifs by TMHMM 2.0 and Phobius [55, 56] (Additional file 1: Fig. S1), respectively. Combining these data, 2085 genes (2.19%) were predicted to encode secreted proteins that have a signal peptide, lack a transmembrane domain, and were predicted as extracellular (Additional file 1: Fig. S1; Additional file 2: Table S1).

**Fig. 1:**
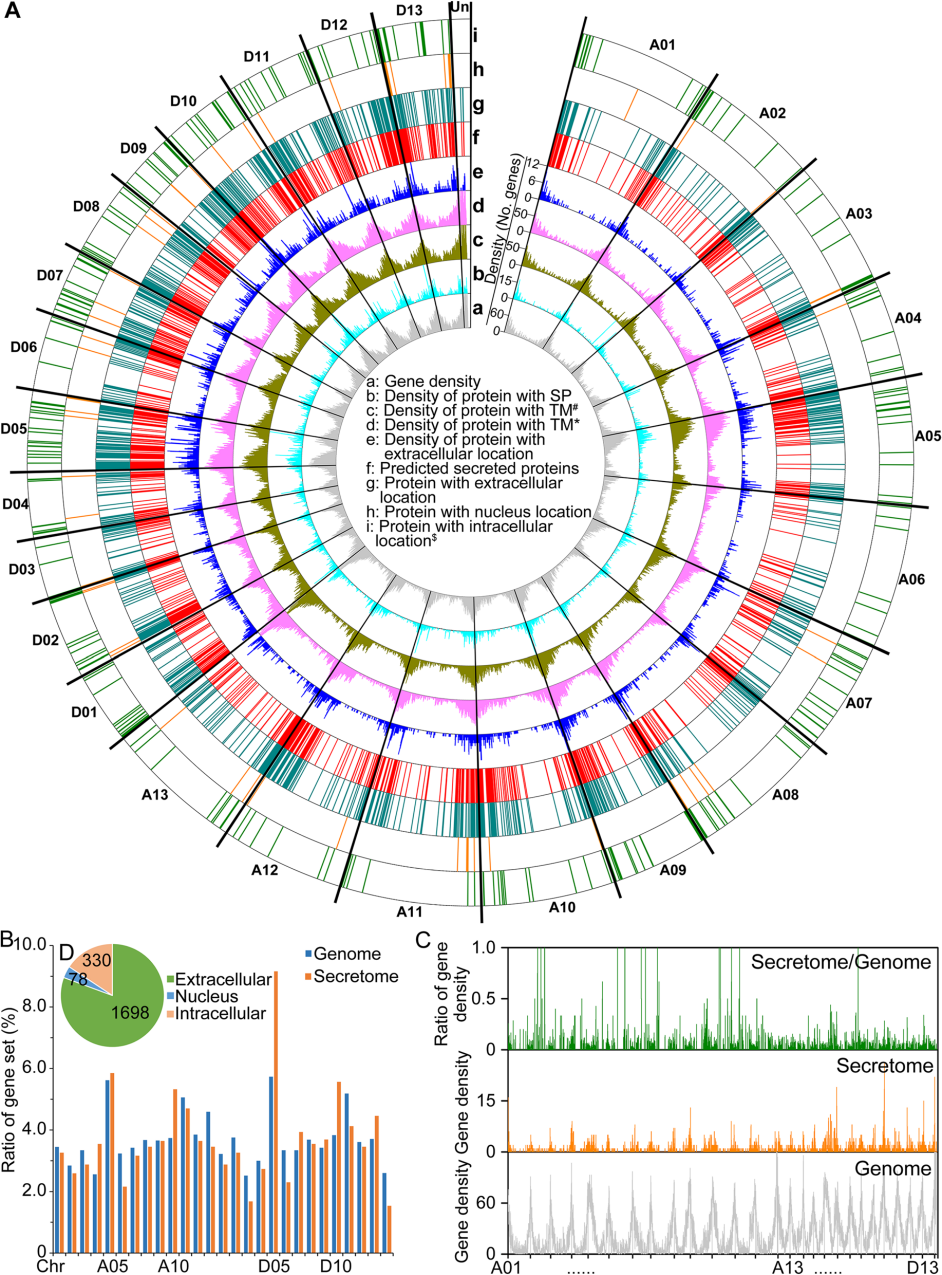
Prediction of genes encoding secreted proteins and their distribution on the chromosomes in the genome of cotton cultivar Zhongzhimian No.2 (ZZM2). **A** Characteristics of predicted secreted proteins and their corresponding gene distribution on the 26 chromosomes in the cotton genome. The density data were calculated by the number of encoded genes using step windows (window = 500 kb, walking step = 100 kb). Secreted proteins were predicted as those with signal peptide (SP), lack of the transmembrane (TM) domain, and the extracellular location. The transmembrane domain was predicted by two tools, TMHMM2.0 [55] and Phobius [56]. The subcellular location of the prediction of secreted proteins was carried out using the plant model of the WolfPsort procedure by (circle e) [54], and the predicted localization during the host–pathogen interaction was predicted using the fungi model (circle g–i). A01–A13 and D01–D13 represent the 13 chromosomes of the A sub-genome and the D sub-genome of the ZMM2 genome, respectively. **B** Ratio of secreted proteins encoded in each chromosome versus the total number of secreted proteins. The statistic of the encoded genes in the genome was set as the comparison group. **C** Comparison of the gene density between the encoded putative secreted proteins and total encoded proteins by step windows. Step window: window = 500 kb, walking step = 100 kb. The top panel represents the ratio that the gene density of encoded secreted proteins versus the gene density of total encoded proteins. **D** Prediction of the localization of the secretome during host–pathogen interactions using the fungi model of WolfPsort [54]

Statistical analyses showed that the predicted gene sequences encoding secreted proteins were mainly of short gene length (≤ 400 aa) (Additional file 1: Fig. S2). Association analysis of encoded proteins within the chromosomes indicated that the distribution of the predicted secretome genes was irregular (Fig. 1A; Additional file 1: Figs. S3 and S4). Certain chromosomes encode fewer proteins relative to the average of 26 chromosomes, but the protein with a higher number of signal peptide and extracellular location (Additional file 1: Figs. S3 and S4), resulting in the differential distribution of secreted proteins among 26 chromosomes (Fig. 1A). The A10 (111 genes), D05 (191 genes), and D10 (116 genes) encode more secreted proteins than other chromosomes (Additional file 1: Fig. S5). Comparison of the secreted proteins of each chromosome relative to the whole secretome further illustrated the irregular distribution of secreted proteins, as the D05 chromosome possesses 5.7% of total encoded genes but houses 9.2% of total encoded secretome (Fig. 1B). Investigation of the gene density by step-wise windows (window = 500 kb, walking step = 100 kb) also illustrated the irregular distribution of secreted proteins among the 26 chromosomes and their enrichment characteristics (Fig. 1C). Among all step-wise windows, the encoded proteins from 31 windows (28 windows belong to A sub-genome) were predicted as secreted protein rich region, which were located in the gene sparse region (Fig. 1C). Moreover, gene density characteristics showed that the secretome has a higher density in the D sub-genome than the A sub-genome (Fig. 1A, C), which may be the result of similar numbers of encoded proteins in the two sub-genomes (A sub-genome, 994 proteins; D sub-genome, 1,059 proteins) but genome size of the D sub-genome is more compact (A sub-genome, 1469 Mb; D sub-genome, 849 Mb) [49]. The destiny of the predicted secretome during host–pathogen interaction was predicted using the fungal model in WolfPsort [54], revealing that 1698 proteins had a similar predicted subcellular localization of the extracellular space, and 78 proteins were predicted to be localized in the nucleus (Fig. 1A, D). Taken together, the genome of ZZM2 encodes a large set of secreted proteins that display an irregular distribution among the chromosomes.

### Analyses of the conservation and divergence of the cotton secretome

**Fig. 2:**
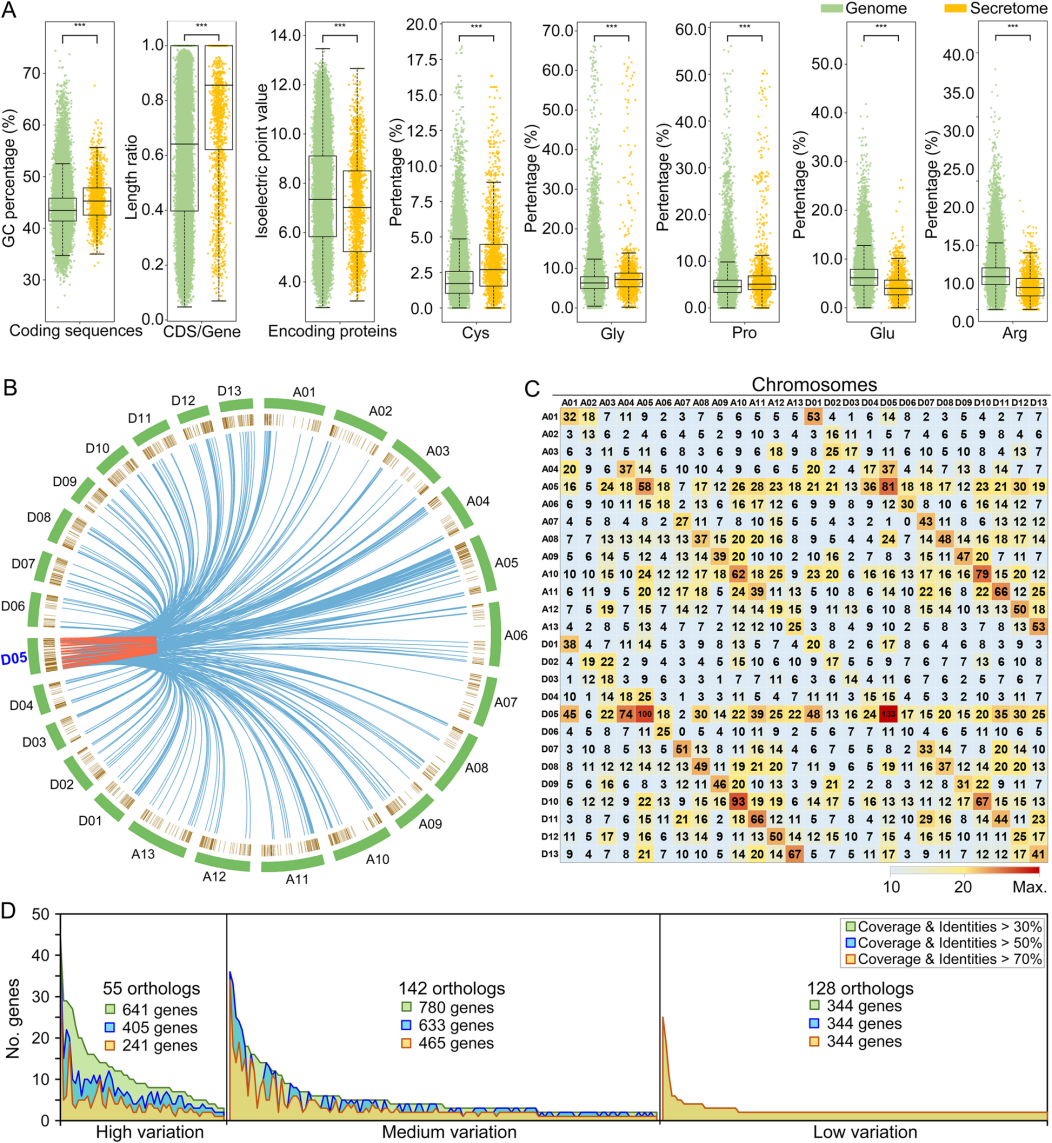
Conservation of the cotton secretome in cultivar Zhongzhimian No.2 (ZZM2). **A** Comparison of the protein properties of the predicted secretome versus the total encoded proteins in the ZZM2 genome. The length ratio of CDS/gene represents the value of coding sequence length compared to the gene length; the value of the gene without intron is 1.0. Asterisks (***) represent statistical significance at *P* < 0.001 based on unpaired Student’s *t*-tests, and Levene’s test was used to assess the homogeneity of variances. **B** Synteny analysis of encoded secreted proteins from the D05 chromosome with those of the other 25 chromosomes. The synteny relationship was constructed by ortholog clustering (both coverage and identities up to 30%) of secreted proteins from the D05 chromosome with other secreted proteins from the other 25 chromosomes, present in blue lines. The red lines represent the self-orthologs of secreted proteins within the D05 chromosome. The outer circle with green blocks represents the 26 chromosomes of the ZZM2 genome, and the inner circle with brown lines represents the secreted proteins encoded in the ZZM2 genome. **C** Matrix representing the gene number of each chromosome with ortholog relationships of the 26 chromosomes. The data in columns but not in rows represent the gene number of each chromosome (top labels) in ortholog clustering (both coverage and identities up to 30%) with the other chromosomes (left labels). **D** Sequence divergence of orthologs detected under different ortholog clustering parameters. The ortholog number was determined by the ortholog clustering with 30% parameters, and the attenuation of total gene numbers among these orthologs was investigated under the 50% and 70% parameters. High variation represents the total gene numbers attenuated from 30 to 50 and 70%, medium variation represents the total gene numbers attenuated from 30 to 50 or 70%, and low variation represents identical sequence under 30%, 50%, and 70% parameters

Plant-secreted proteins are anticipated to mediate multiple responses in their external environment and, as such, may possess different sequences and biochemical properties. Indeed, investigation of the putative secreted proteins clearly indicated that they possess different sequence characteristics compared to those of the total predicted proteins from the ZZM2 genome (Fig. 2A). The members of the secretome contain a higher GC percentage and exhibit reduced introns/intron length (length ratio of coding sequence compared to gene length). The composition of amino acid residues in the predicted secreted proteins also displayed divergence compared to those in the remainder of the genome. Predicted proteins of the secretome were enriched in cysteine, glycine, and proline residues, but not in glutamic acid and arginine residues, resulting in their lower isoelectric point compared to the overall proteins encoded in the ZZM2 genome (Fig. 2A; Additional file 1: Fig. S6). These results clearly revealed differences in the predicted properties of core encoded proteins and the ZZM2 secretome, likely a result of their relatively unique localization in the extracellular space.

Sequence conservation and variation within the secretome of ZZM2 were investigated by examining the relationships among orthologs. From both the coverage and identities of up to 30%, 50%, and 70%, 1765, 1651, and 1416 genes were clustered in 325, 409, and 447 orthologous groups (Additional file 2: Table S2), respectively. In addition to the maximum orthologous sequence that was enriched in more than 40 genes, more than 20% of orthologs (under the 50% parameters) were enriched in sets of five or more genes (Additional file 2: Table S2). Sequence alignment revealed the conservation characteristics among members of the maximum orthologous sequence (Additional file 1: Fig. S7). Furthermore, the syntenic relationship among the encoded secreted proteins was investigated by ortholog clustering (both coverage and identities up to 30%) in each chromosome and in relation with other chromosomes. The results showed the expected high synteny with higher gene synteny pair numbers between the allelic chromosomes in the A and D sub-genomes (Fig. 2B, C; Additional file 1: Fig. S8), as 81 members from A05 chromosome (122 secreted proteins in total) had syntenic pairs with the allelic D05 chromosome (191 secreted proteins in total) (Fig. 2C). In addition, the orthologs of secreted proteins also displayed the comparable high syntenic pairs within chromosomes (red lines) (Fig. 2B, C), as the 62 genes from chromosome A10 (111 genes) or 133 genes from chromosome D05 were present in the self-ortholog clustering analysis (Fig. 2C). These results were further supported by the rigorous ortholog clustering (parameter of 50% or 70%), in which there were highly syntenic pairs within a chromosome or allelic chromosomes (Additional file 1: Fig. S9). Thus, these results suggested that the tandem duplications of genes encoding secreted proteins frequently occurred among chromosomes of ZZM2. Intriguingly, except for the allelic chromosome, the genes encoding secreted proteins also showed high synteny to those present in other chromosomes (Fig. 2B), as the A05 and D05 chromosomes display comparable syntenic pairs as with other chromosomes (Fig. 2C; Additional file 1: Fig. S9), suggesting that segmental duplication also occurred among different chromosomes. These results indicated that a number of the predicted members of the secretome may be conserved within the allotetraploid cotton genome and that some of this conservation may be driven by tandem or segmental duplications. However, there is also a clear sequence divergence within the predicted secretome of the ZZM2 genome. Ortholog clustering with parameters from 30 to 70% revealed attenuated gene numbers of 55 orthologs (30%, 641 genes; 50%, 405 genes; 70%, 241 genes), indicating high variation among these orthologs. Additionally, a second tier of 142 orthologs exhibiting medium variation displayed similar variation (30%, 780 genes; 50%, 633 genes; 70%, 465 genes), while only 128 orthologs exhibited low variation for the same gene numbers under the three clustering parameters (Fig. 2D). Although cotton is an allotetraploid species, and it is anticipated that most genes would be allelic for the two sub-genomes, there are at least 320 unique genes (30% parameters), which cannot be grouped in orthologs (Additional file 2: Table S2). Therefore, while the secretome of the ZZM2 genome reveals some of the expected conservation for an allotetraploid genome, it also exhibits variation in the sequences of orthologs and the presence of unique genes between the two sub-genomes.

### Functional analyses of the ZZM2 cotton secretome

**Fig. 3:**
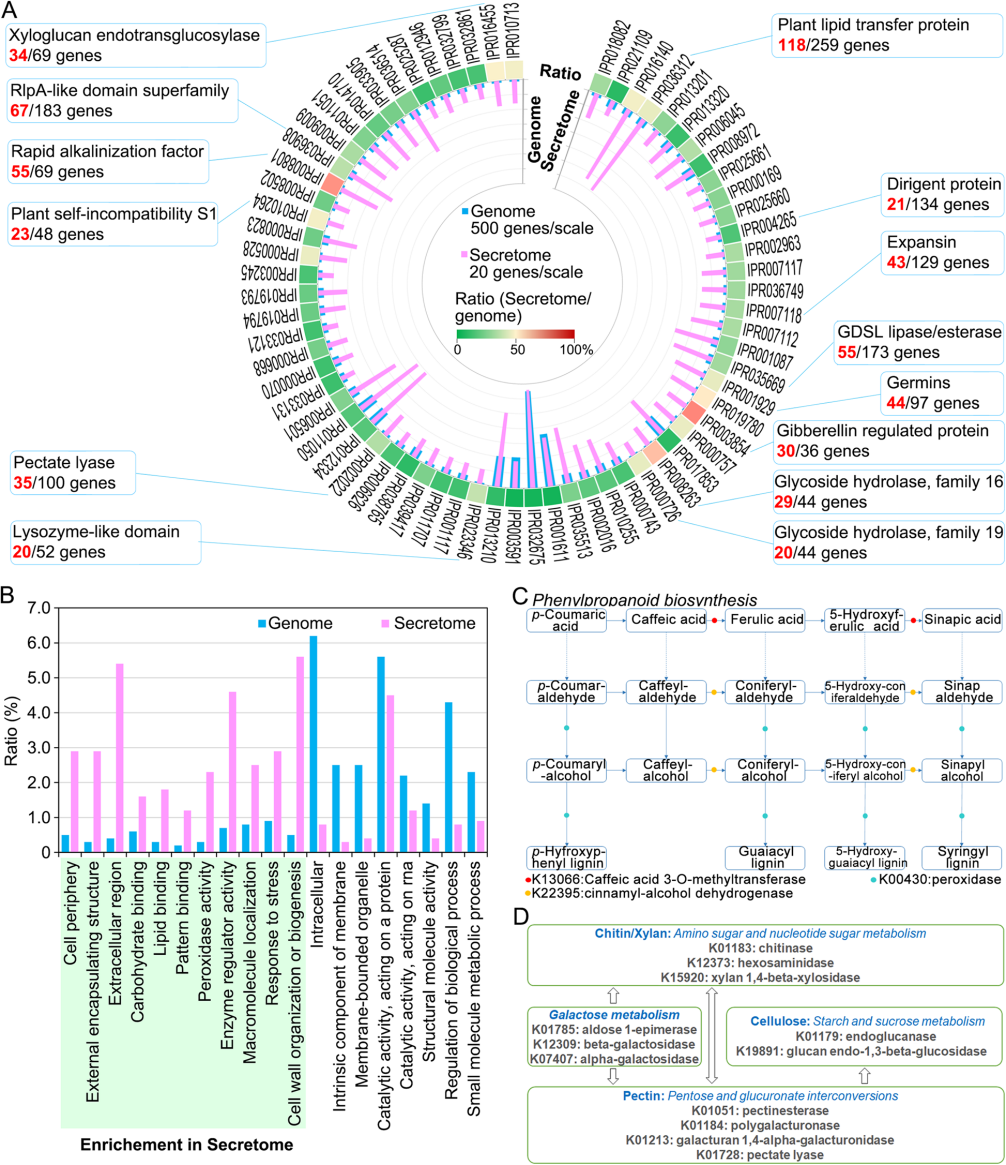
Functional annotation of the secretome from cotton cultivar Zhongzhimian No.2. **A** Functions of the secretome predicted by conserved domains. The conserved domains of secreted proteins were predicted by the Interpro database using InterProScan (https://www.ebi.ac.uk/interpro/), and total predicted proteins within the genome were set as the control. Columns in purple and blue color represent the number of proteins of the indicated conserved domains accession (IPR accession) from the secretome (axis on the right side) and whole genome (axis on the left side), respectively; the scale on the secretome or genome axes represents 500 or 20 genes, respectively. The outer circle with heatmap blocks represents the ratio of secretome versus the predicted proteins of the whole genome in the indicated IPR accession. The blue boxes link to IPR accessions represent the high ratio of indicated conserved domains in the secretome (number in red color) versus the genome (number in black color). **B** Comparison of the Gene Ontology (GO) annotation between predicted encoded proteins from the whole genome versus the secretome. Significant enrichment was determined by a Pearson chi-square test at *P* < 0.001, and the items with a green-colored background represent a significantly higher functional enrichment in the secretome versus those from the whole genome. **C** Enrichment of secreted proteins in the phenylpropanoid biosynthesis pathway. The potential pathways were predicted by the KEGG database (https://www.kegg.jp/), and members with homologs of phenylpropanoid biosynthesis pathway (Accession ID: ghi00940) were selected for conceptualization. **D** Enrichment of secreted proteins with predicted polysaccharide metabolism function. The secreted proteins associating with four polysaccharide metabolism pathways were selected for conceptualization. **E** Statistics of gene numbers with functional annotation of the genome and secretome in GO and KEGG database

The plant secretome includes those proteins that are involved in responding to biological stress, including pathogen attacks. Thus, the functional characteristics of the secretome were predicted using InterPro (conserved domain), Gene Ontology (biological function), and KEGG (function network) (Additional file 2: Table S1). Prediction of the conserved domains revealed that 1495 genes have 443 conserved domains/motifs (IPR accessions) (Additional file 2: Table S3), and 69 IPR accessions contain at least 20 genes (Fig. 3A; Additional file 2: Table S4). Analysis of the functions of conserved domains suggested that the secretome functions in defense responses (dirigent protein, leucine-rich repeat protein, gibberellin regulated protein, and lysozyme-like domain protein), polysaccharide metabolism (as the glycoside hydrolase, pectate lyase, and xyloglucan endotransglucocylase), and cell wall strengthening (expansin, rapid alkalinization factor, plant lipid transfer protein) (Fig. 3A; Additional file 2: Table S4). Gene Ontology annotation further indicated that a portion of the secretome functions extracellularly in defense and in stress responses, since there was a high ratio of secreted protein enriched in carbohydrate binding, peroxidase activity, and response to stress (*P* < 0.05) (Fig. 3B). Finally, KEGG network analysis showed that only 613 genes were matched to 63 pathway accessions (Additional file 2: Table S3). Of these pathway accessions, members of the secretome were highly enriched in the phenylpropanoid biosynthesis pathway and carbohydrate metabolism (Additional file 2: Table S5), which share defense-related functions, especially lignin production, associations with phytoalexin biosynthesis, and polysaccharide metabolism (pectin, cellulose, etc.) (Fig. 3C, D). However, the secretome may present a more complex unknown function in the biological stress response since most members are predicted hypothetical proteins for which there is no predicted biological function (Additional file 2: Table S3). Together, the secretome of the ZZM2 genome shares predicted functions concordant with responses to biological stress in the extracellular space, such as in the defense response and in polysaccharide metabolism.

### Transcriptome analyses reveal a role for the secretome in resistance to *Verticillium dahliae*

**Fig. 4:**
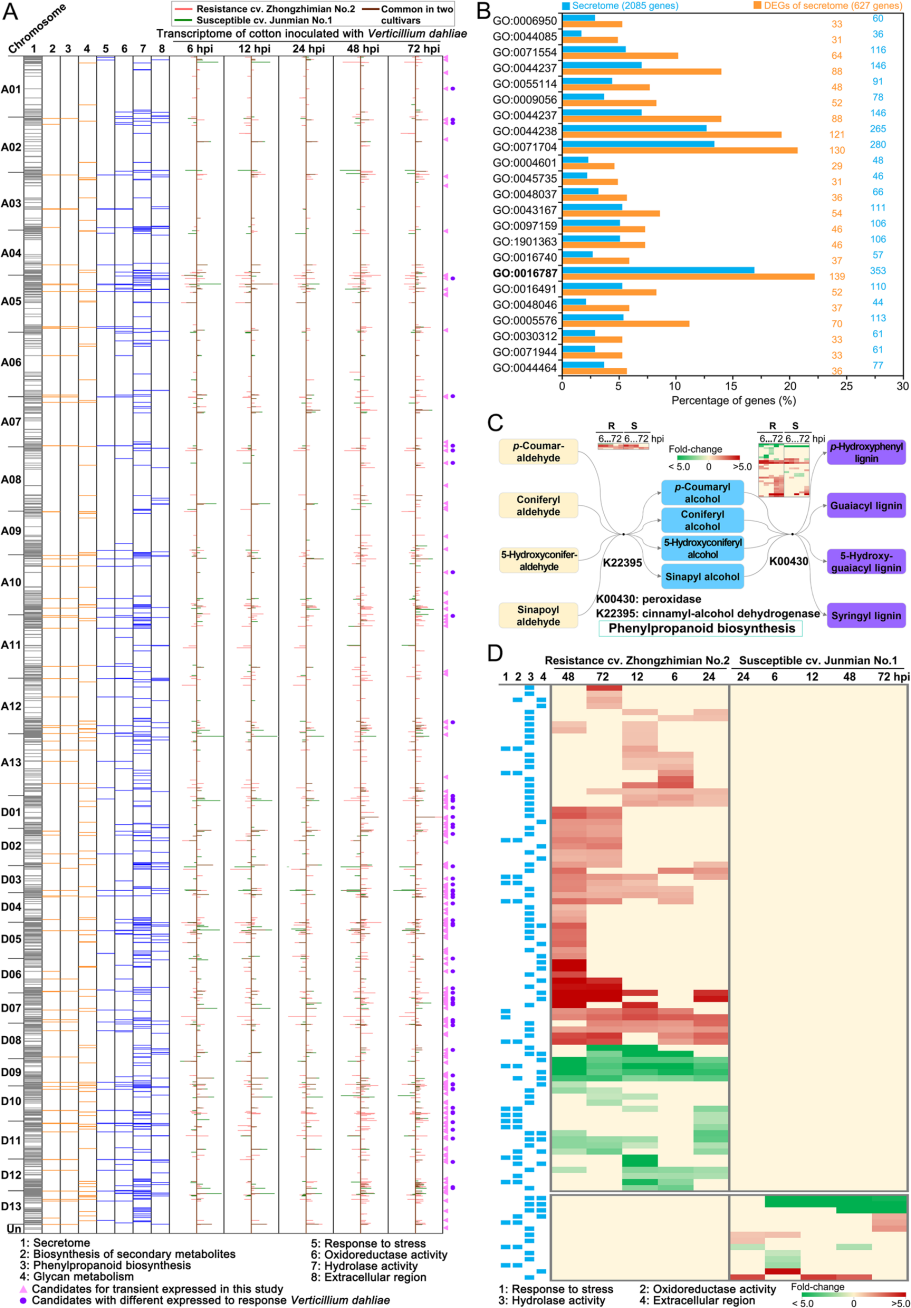
Transcriptome analyses of the secretome of cultivar Zhongzhimian No.2 (ZZM2) in response to *Verticillium dahliae*. **A** Gene expression patterns of the secretome and their functional enrichment in response to *V. dahliae* infection. The resistant cultivar (cv. Zhongzhimian No.2, ZZM2) and susceptible cultivar (cv. Junmian No.1) with the time course (6, 12, 24, 48, 72 h post-inoculation) samples were performed for transcriptome analyses. The filtering parameters of DEGs were |log_2_FoldChange|≥ 1.0 and *P*adj < 0.05. All differentially expressed genes (DEGs) were painted on the chromosomes according to their physical position. The DEGs of the same time point from the resistance cultivar (red columns) and susceptible cultivar (green columns) are shown as overlapping for comparison, and the columns showing “gene response” in both cultivars are shown in brown color. Secreted proteins classified in three KEGG annotation pathways (No. 2–4) and according to four gene ontology (GO) annotations (No. 5–8) are labeled in orange and blue lines, respectively. Pink triangles represent the candidates for functional validation, and the purple dots represent the selected candidates that were differentially expressed in response to *V. dahliae*. **B** Significant catalogs of Gene Ontology (GO) enrichment of DEGs in the ZZM2 secretome in response to the *V. dahliae* infection. The significant categories were selected by the Pearson chi-square test with *P* < 0.05, and the total secreted proteins of the secretome were set as the control. Information on GO categories is listed in Additional file 2: Table S8, and the GO category of hydrolase activity (GO:0016787) is labeled in bold font. **C** The expression pattern of phenylpropanoid biosynthesis-related genes in response to *V. dahliae*. The heatmap representation includes the five secretome members that are differentially expressed and function in the biological process of oxidation (K00430) and 29 members that are differentially expressed and function in the biological process of dehydrogenation (K22395). Letters “R” and “S” represent the resistant cultivar ZZM2 and the susceptible cultivar (cv. Junmian No.1), respectively. **D** Heat map analyses showing expression of the predicted secretome in response to *V. dahliae* in the resistant cultivar ZZM2 or the susceptible cultivar Junmian No.1. Blue boxes (left side) represent four Gene Ontology (GO) annotations

Disease resistance is a component of biological stress responses in the extracellular space. Thus, we employed RNA-Seq-based transcriptome analyses of cotton challenged by *V. dahliae* at a critical infection stage (within 3 days following infection with *V. dahliae*, the pathogen enters the root xylem vessels) [57], to determine the role of the secretome against *V. dahliae.* In total, 627 members of the predicted encoded secretome were differentially expressed (DEGs, |log_2_FoldChange|≥ 1.0 and adjusted *P* < 0.05) in the resistant cultivar (ZZM2) and susceptible (cv. Junmian No.1) cultivar in response to the *V. dahliae* infection at five time points (Additional file 1: Fig. S10A; Additional file 2: Table S6). The physical location of the DEGs on chromosomes showed that many secretome members are specifically induced in ZZM2 during infection (red columns clusters) in A05, A10, A12, and their allelic chromosomes (Fig. 4A). In addition, the function of these DEGs was associated with the phenylpropanoid biosynthesis pathway, carbohydrate metabolism, and extracellular stress response (Fig. 4A). GO analysis further showed that the functions of DEGs were significantly enriched in extracellular stress response versus the encoded proteins of the secretome (*P* < 0.05), including the functions of oxidoreduction and hydrolase activity (Fig. 4B; Additional file 2: Table S7), and may yield unique responses in the resistant versus the susceptible cultivars (Additional file 1: Fig. S11). Additionally, the secretome members involved in phenylpropanoid biosynthesis were also strongly responsive during *V. dahliae* infection. Five members of the peroxidases (K00430) and 29 members in the cinnamyl-alcohol dehydrogenase function were all induced and participated in lignin biosynthesis (Fig. 4C; Additional file 2: Table S8). Correspondingly, histochemical analysis of lignin in stem cross-sections showed higher lignification in the xylem vessels and interfascicular fibers in the resistant cultivar ZZM2 than in the susceptible cultivar Junmian No.1 (Additional file 1: Fig. S12). Finally, the resistant cultivar employed more members of the secretome than the susceptible cultivar in response to *V. dahliae* infection and was investigated using the transcriptome data. During infection, the numbers of secretome members differentially expressed in the resistant cultivar were higher than in the susceptible cultivar at each sampling point, especially at 48 h post-inoculation (404 genes in the resistance cultivar versus 144 genes in the susceptible cultivar) (Additional file 1: Fig. S10A). GO enrichment revealed that these genes in the resistant cultivar have multiple functions (extracellular stress response, hydrolase activity, carbohydrate metabolic process, etc.) during *V. dahliae* infection (Additional file 1: Fig. S13A). Moreover, among the 627 DEGs, 257 were expressed in the resistant but not susceptible cultivar in response to *V. dahliae* (Additional file 1: Fig. S10B), and the majority of these DEGs were involved in the extracellular stress response of hydrolase activity, oxidoreductase activity, and were extracellular (Fig. 4D; Additional file 1: Fig. S13B; Additional file 2: Table S9). In addition, 92 and 34 genes were co-expressed at all sampling points in the resistant or susceptible cultivar following inoculations with *V. dahliae* (Additional file 1: Fig. S10C), respectively. Of these genes, 29 members were co-expressed in the resistant cultivar but were not induced in the susceptible cultivar. Four of those that were significantly upregulated shared homology with genes involved in defense responses, including those encoding pathogenesis-related protein 4 and cell wall inhibitor of fructosidase 1 (Additional file 2: Table S10). Unexpectedly, nearly all of them (25 genes) were downregulated and had functions in signal responses and catalytic activity (Additional file 2: Table S10). Together, the results of the transcriptome analysis strongly suggested that cotton employs its secretome to enhance the extracellular stress response, thus contributing to resistance against *V. dahliae*.

### The cotton secretome plays important roles in the immunity response

**Fig. 5:**
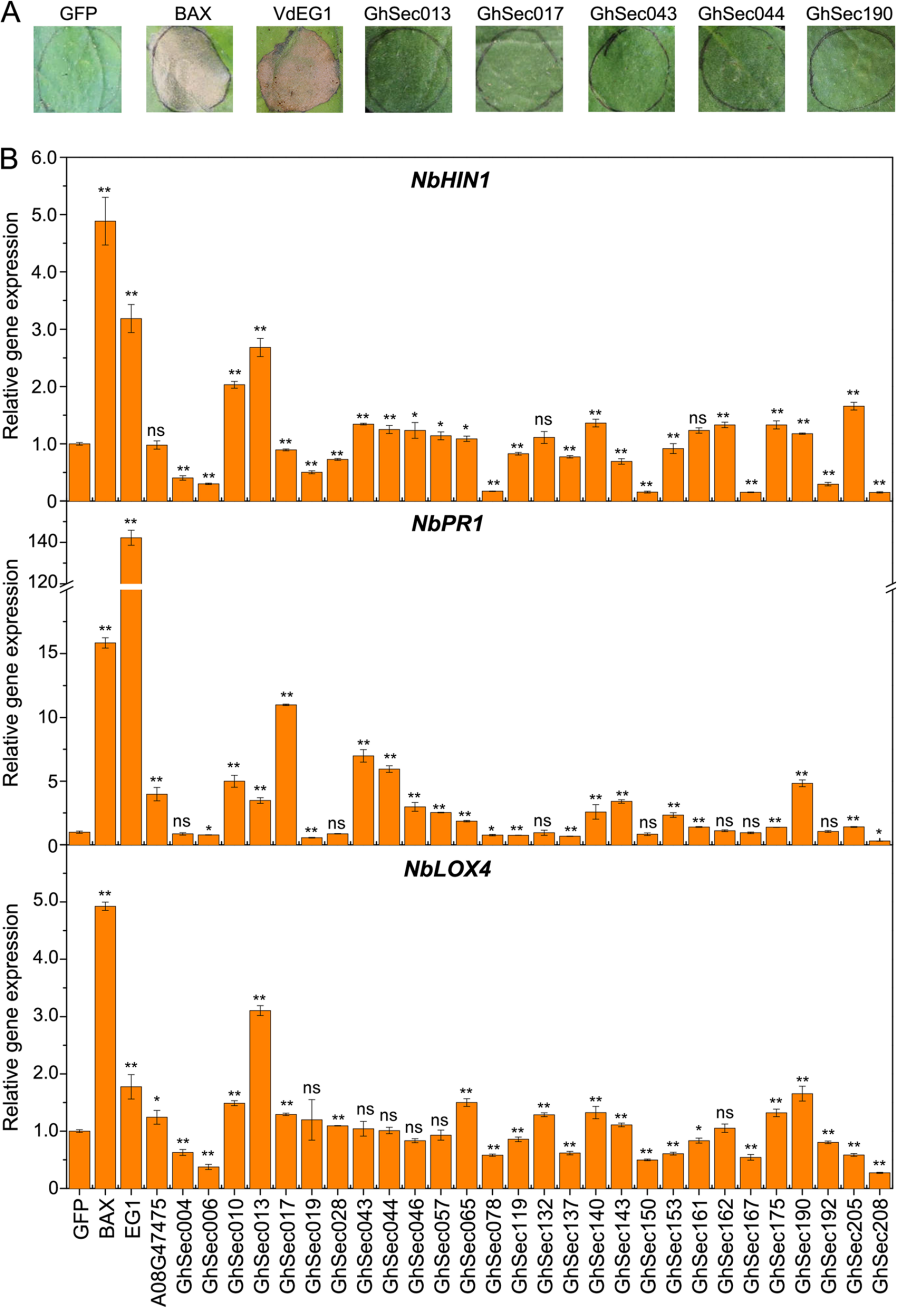
Members of the Zhongzhimian No.2 (ZZM2) secretome activate the plant immune responses. **A** Analyses of induction of cell death in 4-week-old *N. benthamiana* leaves that were infiltrated with constructs expressing 213 members of the secretome of ZZM2. Cell death was examined after 8 days. The PAMP endoglucanase VdEG1- and the Bcl-2-associated X protein (BAX) were used as positive controls; green fluorescent protein (GFP) was used as a negative control. The figure represents selected phenotypes following the infiltration of five members of the ZZM2 secretome: GhSec013, GhSec017, GhSec043, GhSec044, and GhSec0190. **B** Detection of transcripts of defense response genes related to the immunity marker gene *NbHIN1*, salicylic acid signaling marker gene *NbPR1*, and jasmonic acid signaling marker gene *NbLOX4* by reverse transcription-quantitative PCR (RT-qPCR). The transcripts were detected in 4-week-old *Nicotiana benthamiana* leaves 2 days after agro-infiltration with the 18 random selective secretome members BAX, VdEG1, and the cotton A08G47475 (thionin protein) used as positive controls to induce the *N. benthamiana* immunity response. The RT-qPCR experiments were performed three times. Error bars represent standard errors. Asterisks * (*P* < 0.05) and ** (*P* < 0.01) indicate a significant difference relative to the agro-infiltration GFP control in unpaired Student’s *t*-tests

The secretome plays critical roles in homeostasis, immune response, development, proteolysis, adhesion, and in the extracellular matrix [58, 59]. We collected the representative and consolidated secretome members according to ortholog clusterings of the secretome, resulting in the selection of 645 members from the total of 2085 in the original prediction (many members are allelic due to the allotetraploid genome or duplicated). These were filtered to 589 members based upon the presence of a transmembrane domain (predicted by TMHMM2.0) at their *N*-termini (probable overlaps with the signal peptide) and further to 559 members that were without a transmembrane domain and a signal peptide (Phobious). The 559 members were further narrowed to 225, based on the extracellular location score (> 50% probability) and arrived at 213 representative secretome members by filtering the length to up to 400 amino acid (aa) residues (Additional file 1: Fig. S14; Additional file 2: Table S1). To determine whether these 213 secretome members can activate immunity, the cell death-inducing activity of these gene products was examined by transient expression assays in 6-week-old *Nicotina benthamiana* leaves. Unlike the positive control Bcl-2 associated X protein (BAX) or pathogen-associated molecular patterns (PAMP) endoglucanase (VdEG1) [11], which induce obvious cell death at 4 days after agro-infiltration, agro-infiltration assays of all 213 secretome members showed that none caused the cell death phenotype until 8 days after agro-infiltration (Fig. 5A). To further analyze the potential role of the secretome members to induce immunity that is not associated with cell death, the expression of cotton defense response genes was determined at 2 days after agro-infiltration of 28 randomly selected members in *N. benthamiana* leaves, including the immunity marker gene *NbHIN1*, the salicylic acid marker gene *NbPR1*, and the jasmonic acid marker gene *NbLOX4*. As expected, several members have the ability to induce the expression of these defense response marker genes after transient expression in *N. benthamiana* leaves, including upregulation of *NbHIN1* (12 members, several typical members of GhSec010 < hypothetical protein > , GhSec013 < adenosine kinase 2-like > , GhSec205 < rapid alkalinization factor > etc.), *NbPR1* (15 members, several typical members of GhSec017 < gibberellin-regulated protein 14-like precursor > , GhSec043 < classical arabinogalactan protein 5 > , GhSec044 < hypothetical protein >), and *NbLOX4* (10 members, several typical members of GhSec10, GhSec13, GhSec190 < hypothetical protein > , etc.), in relation to the positive controls of BAX, VdEG1, and cotton A08G47475 (thionin protein) (Fig. 5B). All three defense marker genes were upregulated when seven members of GhSec010, GhSec013, GhSec065 (hypothetical protein), GhSec140 (germin-like protein), GhSec143 (GDSL esterase), GhSec175 (hypothetical protein), and GhSec190 were transiently expressed in *N. benthamiana* leaves (Fig. 5B). Together, these results strongly indicated that the secretome encoded by cotton has the ability to induce the immune responses.

### The cotton secretome confers Verticillium wilt resistance

**Fig. 6:**
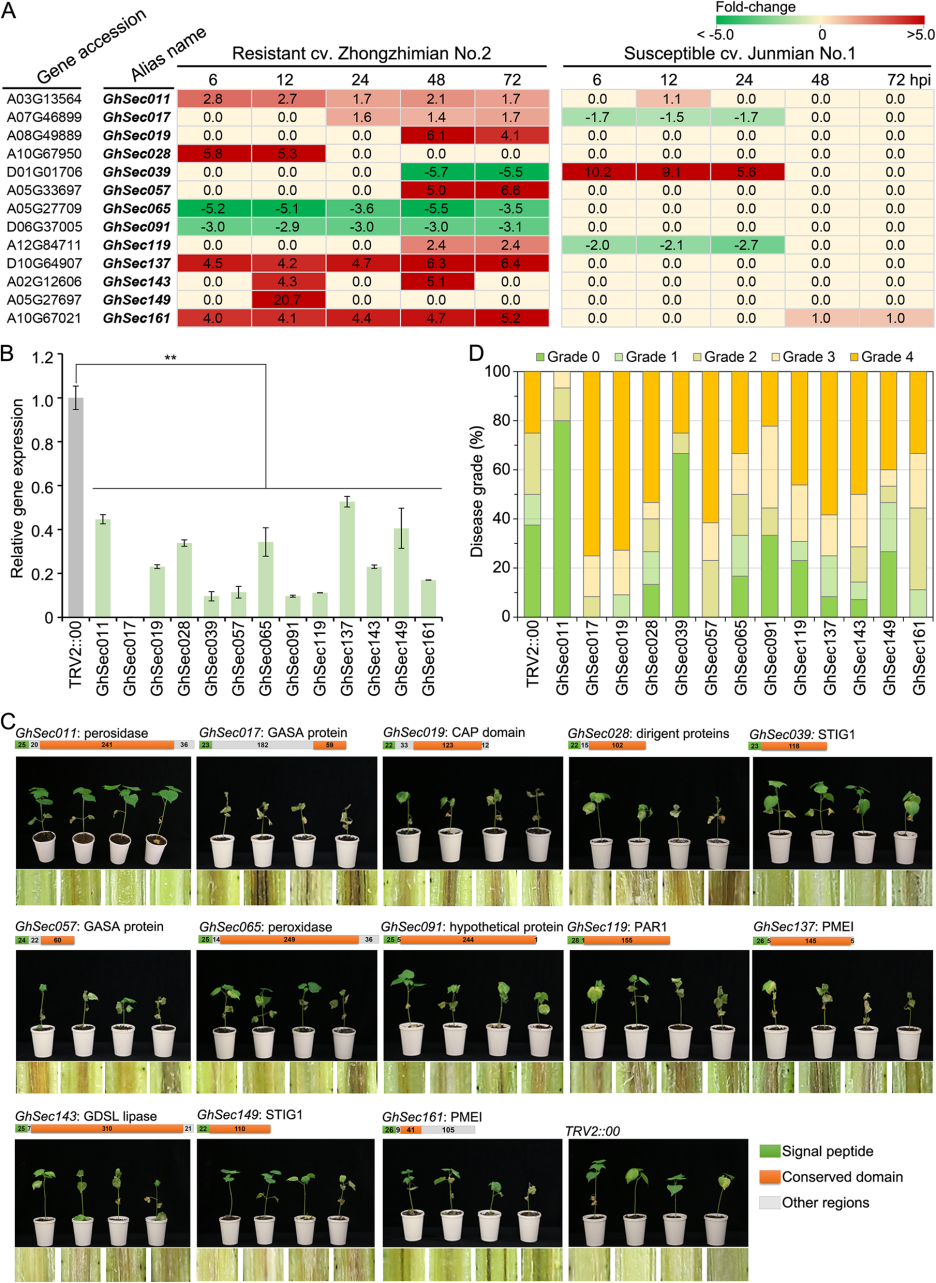
Silencing of cotton secretome-encoding genes by virus-induced gene silencing (VIGS) affects resistance to *Verticillium dahliae*. **A** Expression patterns of 13 selected secretome members in *Gossypium hirsutum* resistant cultivar Zhongzhimian No.2 (ZZM2) and susceptible cultivar Junmian No.1 after inoculation with *V. dahliae* strain Vd991. Values (log_2_ fold change) represent the averages from three biological replicates. Transcript expression data are from the indicated cultivars at the different time points after inoculation with *V. dahliae*. Green shading indicates downregulation, and red shading indicates upregulation. **B** Silencing of selected secretome members in cv. Zhongzhimian No.2 by virus-induced gene silencing (VIGS) affects resistance to *V. dahliae*. Approximately 14 days after the VIGS procedure in 3-week-old ZZM2 plants, the gene-silenced and wild-type (WT) plants were inoculated with 5 mL of *V. dahliae* strain Vd991 conidial suspension (5 × 10.^6^ conidia/mL) or sterile water (mock) using a root-dip method. Experiments consisted of three replicates of 12 plants each arranged in a complete random block design. The Verticillium wilt phenotypes of wilting leaves and vascular discoloration were photographed 4 weeks after inoculation. Infiltration with the empty vector pTRV2 (TRV2:0) served as a positive control. **C** Evaluation of the disease symptoms in gene-silenced plants inoculated with *V. dahliae*. The disease ratings were classified as grade 0 (healthy plants), grade 1 (0–25% leaves wilting), grade 2 (25–50% leaves wilting), grade 3 (50–75% leaves wilting), and grade 4 (75–100% leaves wilting). The ratings were conducted with 12 cotton seedlings at 3 weeks post-inoculation with three replicates. All the disease index value displays significant change among the gene-silenced plants compared to the positive CLA1-silenced cotton plants (*P* < 0.01). **D** The silencing efficiency of 13 selected secretome members was determined by RT-qPCR analysis. The cotton *GhUbiquitin* gene was used as an endogenous control. CK represents the control infiltration with the empty vector pTRV2 (TRV2:0). The means and standard errors from three biological replicates are shown. Asterisks ** indicate significant differences (*P* < 0.01)

To further examine the role of the secretome in Verticillium wilt resistance in cotton ZZM2, we selected 13 secretome members in which the expression pattern was associated with the Verticillium wilt resistance, i.e., opposite expression patterns between resistant (ZZM2) and susceptible (cv. Junmian No.1) cultivars (Fig. 6A). For instance, the expression of *GhSec137* (encodes pectin methylesterase inhibitor) was strongly upregulated in the resistant cultivar (ZZM2), but its expression was not affected in the susceptible cultivar (cv. Junmian No.1) after inoculation with the highly virulent *V. dahliae* strain Vd991 (Fig. 6A). Next, tobacco rattle virus (TRV)-based virus-induced gene silencing (VIGS) was performed to assess the function of the 13 selected secretome members from cv. ZZM2. The RT-qPCR analyses indicated that each of the secretome member genes was significantly decreased (Fig. 6B). Subsequently, the silenced plants were challenged with *V. dahliae* after all the *CLA1*-silenced cotton plants presented an albino phenotype on their newly emerged leaves. The results showed that of the secretome member genes assayed, 11 of the secretome gene silenced plants exhibited symptoms of Verticillium wilt compared with the positive *CLA1*-silenced cotton plants after inoculation with *V. dahliae* strain Vd991 and conversely of *GhSec011* and *GhSec039* silenced plant displayed higher resistance than the positive *CLA1*-silenced cotton plants (Fig. 6C). Evaluation of the disease symptoms by leaf wilting ratio analyses confirmed that these genes conferred Verticillium wilt resistance (11 secretome members) or susceptibility (*GhSec011* and *GhSec039*) in cotton cv. Zhongzhimian No.2 (Fig. 6D). In addition, ortholog analysis showed that three Verticillium wilt resistance candidates (GhSec017, GASA protein; GhSec065, peroxidase; GhSec091, hypothetical protein) belong to a unique group in the cotton secretome (Additional file 2: Table S11), and other associated candidate orthologs belong to multiple groups that exhibit sequence divergence (Additional file 1: Fig. S15; Additional file 2: Table S11), which indicated that these candidates are important members of arm-race between cotton and *V. dahliae* interactions. Taken together, these results strongly suggested that the secretome plays a critical role in cotton Verticillium wilt resistance.

## Discussion

The interface between a host plant and pathogen is the initial battlefield where there occurs a “joust” for life or death [2], and the secretome plays a critical role at this interface in the maintenance of cell wall structure, sensory functions, communication, etc. [3, 18]. The roles of the secretome during plant infection have been well studied from the pathogen side and include the degradation of host cell walls, manipulating immunity, scavenging host reactive oxygen species, acquiring nutrition, etc. [4, 5]. Host plants also have employed the secretome to activate defense responses for restricting pathogen proliferation [18], but the knowledge of the host secretome in disease resistance and their functions is limited. In this study, we employed the reference genome of Verticillium wilt-resistant cotton cultivar ZZM2 [49] to examine its secretome in relation to its role in Verticillium wilt resistance in cotton. Bioinformatics-driven analyses showed that ZZM2 encodes 2085 putative secreted proteins, and these were enriched in responses to stress as may occur in the extracellular space (Fig. 1). Transcriptome analysis corroborated our hypothesis that cotton employs the secretome for resistance against the *V. dahliae* infection (Fig. 4); the immune response was activated to transiently express select secretome members (Fig. 5). This feature was verified by the gene silencing assays in which ZZM2 displayed higher susceptibility to *V. dahliae* after suppressing the gene expression level of selected secretome candidates (Fig. 6), suggesting that secretome members confer Verticillium wilt resistance in cotton.

The plant secretome plays important roles in homeostasis, immune response, development, proteolysis, adhesion, extracellular matrix organization, and communication between different cells, and its composition changes in response to various stresses and environmental stimuli [18, 58]. Thus, the secretome of several plant species has been investigated in planta, and these studies have revealed their diversity in function in stress responses, especially in host plant–pathogen interactions [20, 60, 61]. Host plants release extracellular vesicles (EVs) that contain various types of bioactive substances, including proteins, nucleic acids, and lipids, to function in plant–microbe interactions [20, 61] and in the defense responses against pathogens. For instance, sunflower releases important secreted defense proteins (PR-4, PR-5, PR-6, PR-9, PR-14, proteases, PMR5, Gnk2 antifungal protein, GDSL lipase acylhydrolases, etc.) by EVs when infected by *Sclerotinia sclerotiorum*, resulting in inhibited pathogen growth and/or cell death [62]. Similarly, pepper secretes proteins with a signal peptide present in defense- and stress-related proteins, proteases and protease inhibitors, and cell wall structural proteins, to enhance the ability against *Phytophthora capsica* [63]. In our study on the predicted secretome of resistance cultivar ZZM2, we observed functional characterization as a defense response (pathogenicity-related proteins, oxidation-related proteins, etc.) and cell wall strengthening (Fig. 3), and the corresponding components involved in extracellular stress response (oxidoreduction, hydrolase activity) and cell wall remodeling (such as phenylpropanoid biosynthesis) were significantly enriched in the transcriptome of ZZM2 challenged by *V. dahliae* (Fig. 4). Moreover, several selected secretome members displayed critical roles in the Verticillium wilt resistance (Fig. 6C), and the orthologs of several other crops have been proven to play critical roles in disease resistance, as the dirigent proteins have been demonstrated to play significant roles in the plant–pathogen interactions [64] and as also the PMEIs in several hosts [65–68]. In addition, as with the Cys-rich repeat protein 1 (CRR1) that plays a critical role in Verticillium wilt resistance [48], most of the secretory proteins have orthologs in other cotton genomes with different levels of Verticilium wilt resistance (Additional file 2: Table S12). CRR1 is expressed at significantly higher levels in the resistant cultivar relative to the susceptible cultivar (Fig. 6A), suggesting a quantitative defense stimulation by the cotton secretome in Verticillium wilt resistance. In a previous study in *Aabidopsis thaliana*, secreted proteins that participated in these biological processes (peroxidases, serine carboxypeptidase, galactosidase, germin-like protein, etc.) were shown to play critical roles in the defense against *Verticillium longisporum* [69]. Additionally, several secreted proteins contribute to resistance against *V. dahliae*, including chitinase 28 (Chi28) and CRR1 [48], pectin methylesterase inhibitor 3 (GhPMEI3) [70], and the subtilase-like protein GbSBT1 [37]. Thus, the plant secretes a wide range of molecules into the extracellular space which play crucial roles in signaling, development, and stress responses. The cotton secretome also contains members with the typical characteristics described for a role in defense and contribute to Verticillium wilt resistance (such as proteins involved in defense response and cell wall strengthening) during infection by *V. dahlia*e.

The plant secretome contains many components to regulate a variety of plant immune responses, including ROS production, transcriptional reprogramming of genes involved in immunity, and the hypersensitive response [71]. These proteins include proteases (Rcr3, Pip1, CP2, etc.), chitinase, cystatins, peroxidase, and defensins necessary to protect themselves against pathogens or to mediate recognition of pathogen virulence factors, which leads to the induction of defense responses [22, 60, 72]. For instance, tomato can secrete protease Rcr3 in its apoplast, which is recognized by the Cf-2 receptors to mediate the induction of defense responses for resistance against *Cladosporium fulvum* [73, 74]. The plant defensins can block the function of the fungal H + -ATPase, leading to cell death, or induce the production of reactive oxygen species (ROS) and nitric oxide [75]. In our study, we confirmed that several selected members can induce immune responses as determined by the activation of defense response marker genes (Fig. 5B). A clear example of a plant-secreted protein involved in host immunity was identified in *T. intermedium* [29]. The secreted aspartic protease TiAP can interact with the pathogen chitin deacetylase which promotes the liberation of chitin fragments and further activates the host immune system [29]. Based on our results, the secretome encoded by cotton similarly acts as a front line of defense and plays a pivotal role in disease resistance.

The plant cell wall, a dynamic and complex structure surrounding every plant cell, has been demonstrated to have a significant impact on disease resistance and/or on abiotic stresses, and has also emerged as an essential component of plant monitoring systems, thus expanding its function as a passive defensive barrier [76, 77]. For instance, remodeling of primary and secondary cell walls by impairing the function of cellulose synthase (CESA) genes has a specific impact on pathogen resistance and tolerance to abiotic stresses, as shown in the *Arabidopsis* irregular xylem cell wall mutants defective in (CESA) subunits required for secondary cell wall formation. These show enhanced resistance to different pathogens, including the necrotrophic fungi *Plectosphaerella cucumerina* and *Botrytis cinerea*, the vascular bacterium *Ralstonia solanacearum*, and the vascular fungus *Fusarium oxysporum* [78, 79]. Lignin is one of the main components of plant cell wall, and lignin biosynthesis represents a response to a variety of biotic and abiotic stresses [80]. Increased accumulation of lignin can provide a basic barrier against pathogen spread and reduces the infiltration of fungal enzymes and toxins into plant cell walls [81]. In *Arabidopsis*, the cinnamyl alcohol dehydrogenases were highly expressed in roots with strong lignification and induced by pathogens invading *A. thaliana* [82], indicative of lignin as a barrier against pathogens to increase disease resistance. In our study, bioinformatics analyses revealed that the cotton secretome functions in cell wall strengthening, including the polysaccharide metabolism and cell wall biosynthesis (Fig. 3A, C). In particular, the functional annotation of cotton secretome revealed enrichment of the pathway of phenylpropanoid metabolism that is important in lignin biosynthesis (Fig. 3C). Transcriptome analysis further supported a function of cell wall biosynthesis, and those genes identified as involved in lignin biosynthesis were strongly activated in the resistant cultivar ZZM2 compared to the susceptible cultivar Junmian No.1 when challenged with *V. dahliae* (Fig. 4C, D). Previous studies have suggested that lignin biosynthesis plays a critical role in the cotton and tomato Verticillium wilt resistance [83–88], a trend that was also apparent in the analysis of the cotton secretome in this study (Figs. 3 and 4). The cotton lignin biosynthetic gene *Gh4CL30* regulates lignification and contributes to Verticillium wilt resistance [88], and the cotton laccase gene *GhLAC15* enhances Verticillium wilt resistance via an increase in defense-induced lignification and lignin components in the cell walls of plants [86]. Together, these results strongly suggested that the cotton secretome plays a critical role in cell wall biosynthesis, especially in lignin biosynthesis.

## Conclusions

In conclusion, we employed bioinformatics-driven approaches for secretome prediction using the genome of Verticillium wilt resistance cotton cultivar ZZM2. The predicted secretome contained functions consistent with its role in response to biological stress and within the extracellular space, involving immune responses and the creation of a defensive barrier by cell wall strengthening. Transcriptome analysis and gene function validation further revealed that the secretome plays a critical role in the resistant cultivar ZZM2 against the infection of *V. dahliae* through the activation of immune responses and plant cell wall lignification. These findings will help understand the role of the cotton secretome in the Verticillium wilt resistance and identify the Verticillium wilt resistance genes in follow-up studies.

## Methods

### Plant and microbe materials

The *V. dahliae* wild-type strain Vd991 (highly virulent isolate from *Gossypium hirsutum* from Jiangsu Province in China) [89] was cultured on potato dextrose agar (PDA, 200 g potato, 20 g glucose, and 15 g agar per liter) for 5 days at 25 °C. The resistant cotton cultivar (*Gossypium hirsutum* cv. Zhongzhimian No.2, ZZM2) and susceptible cultivar (*Gossypium hirsutum* cv. Junmian No.1) seedlings were grown at 25 °C for 3 weeks for virulence assays. Tobacco (*Nicotiana benthamiana* LAB) seedlings were grown at 25 °C for 4 weeks for transient expression experiments. Both cotton and tobacco plants were grown in a greenhouse with a 14-h light/10-h dark photoperiod. *Agrobacterium tumefacien*s GV3101 was cultured in Luria–Bertani (LB) medium (10 g Tryptone, 10 g NaCl, and 5 g yeast extract in 1000 mL total volume of deionized water) at 28 °C for transient expression experiments in plants.

### Bioinformatics for secretome prediction

The putative secreted proteins encoded in the resistant cotton cultivar ZZM2 genome were identified using the combination of four programs, as described previously [8]. The WoLF PSORT software (plant model) was used for the subcellular localization of all predicted proteins [54]; signal peptides and signal peptide cleavage sites of putative extracellular proteins were predicted using the SignalP software (version 5.0; d-Score cutoff set to 0.500) [53]; the TMHMM 2.0 [90] and Phobius [56] software were employed to identify the transmembrane domain. The protein sequences containing a signal peptide but lacking transmembrane domains were identified as secreted proteins. The gene density was calculated in 100-kb windows along the length of the chromosomes in the cotton genome.

### Ortholog clustering

The ortholog groups among the encoded proteins of the ZZM genome were clustered using two strategies in OrthoMCL [91]. Pairwise sequence similarities between all input protein sequences were calculated using all-by-all BLASTP (parameters: *E*-values < 1e − 25; match length and identities were both 30%, 50%, and 70%); subsequently, a Markov clustering algorithm was applied with an inflation value (2I) of 1.5 (default value in OrthoMCL) for defining ortholog cluster structure. The pairwise matches from the BLAST results were clustered using the clustering application Hcluster_sg [92] for the orthologs among the encoded proteins from the ZZM2 sequenced genomes. The synteny of orthologs among chromosomes were drawn by the Circos program using their physical position on chromosomes [93].

### Transcriptome analysis

Three-week-old seedlings of the resistance cotton cultivar Zhongzhimian No.2 and susceptible cultivar Junmian No.1 were gently uprooted, washed, and dipped into 1 × 10^7^ conidia/mL suspension (5 mL per seedling) of *V. dahliae* for 10 min. Three independent replicates each consisting of 12 plants were inoculated for each treatment, and the samples were collected at 6, 12, 24, 48, and 72 h after inoculation; the seedlings treated with sterile distilled water were controls. Total RNA was extracted using an RNA Purification Kit (Tiangen, Beijing, China) and prepared for sequencing with three biological replicates for each sample. Genomic DNA was removed by DNase treatment, and rRNA was removed by Ribo-zero™ rRNA Removal Kit (Epicenter, USA). Strand-specific sequencing was performed on an Illumina HiSeq 2000 platform, which generated 150 bp paired-end reads. Raw data were processed through in-house perl scripts to obtain clean reads. The clean reads were obtained by removing the adapter and low-quality reads (quality score > Q20). The clean reads were mapped onto the reference genome of *G. hirsutum* cv. Zhongzhimian No.2 (GenBank: JAMQUR010000000) by Tophat2 (v2.0.9) [94] and Bowtie 2 (v2.2.9) [95]. A total of 12 samples were selected for sequencing, including Vd991 inoculated on cv. Zhongzhimian No.2 and cv. Junmian No.1 at 6 h, 12 h, 24 h, 48 h, and 72 h as the treatment group and non-inoculated as the control. Fragments per kilobase of the transcript, per million mapped reads (FPKM) was used to determine expression values. Cuffdiff (v2.1.1) was used to calculate the FPKM of genes in each sample [96]. The fold change in gene expression value was calculated by FPKM treatment/FPKM control. Transcripts were identified as differentially expressed between treatments and controls with the parameters of greater than a twofold change and an adjusted *P*-value < 0.05.

### Functional annotation

Annotations of the total predicted proteins and the secretome were performed with the following programs. Putative functional annotations were assigned using BLASTP to identify the best homologs in the databases of nr, eggnog [97], and InterProScan (incorporated InterPro, GO, and KEGG pathway annotation) [98]. The DEGs were analyzed using Gene Ontology (GO) analysis in the GO-seq package and Kyoto Encyclopedia of Genes and Genomes (KEGG) analysis [99] and the KEGG Orthologous (KO)-Based Annotation System (KOBAS) was employed to explore their biological roles. Significant GO catalogs of the differentially expressed orthologs were selected by the Pearson chi-square test (*P* < 0.05) using the WEGO tool [100].

### Transient expression

The selected genes for transient expression analysis were amplified from the cDNA of ZZM2 plant seedlings or synthesized (Generalbiol, Anhui, China) in the cases where they could not be obtained from the cDNA samples and cloned separately into the PVX vector pGR107 with the ClonExpress II one-step cloning kit (Vazyme, Nanjing, China) according to the manufacturer’s instructions. The recombinant plasmid was transformed into *A. tumefaciens* strain GV3101. *A. tumefaciens* carrying the selected genes were grown in LB medium at 28 °C overnight. The bacteria were harvested and washed in a salt solution containing 10 mM MgCl_2_, 10 mM morpholineethanesulfonic acid (MES), and 200 mM acetosyringone, pH 5.6, and resuspended to an optical density at 600 nm (OD600) of 0.8 for the assays of cell death induction. The transient expression assays were performed using 4-week-old *N. benthamiana* plant leaves injected with the coding sequences of the Bcl-2-associated X protein (BAX) and VdEG1 as-positive controls and the coding sequence of green fluorescent protein as a negative control. Induction of cell death was monitored at 4 days after agro-infiltration on the leaves.

### Gene expression analysis

The transient expression samples were collected at 2 days after agro-infiltration for the analyses of the expression of resistance-related genes in *N. benthamiana* leaves. Total RNA was extracted from the collected samples using a Plant RNA Purification Kit (Tiangen, Beijing, China). cDNA was prepared using M-MLV Reverse Transcriptase and RT-qPCR analyses were conducted using the SYBR Premix Ex Taq kit (Takara) on a QuantStudio 6 Flex Real-Time PCR System (Applied Biosystems, Foster City, CA). The *N. benthamiana* elongation factor 1-α (*NbEF-1α*) gene was used as an internal control to normalize the variance among samples. PCR conditions consisted of an initial denaturation step at 95 °C for 10 min, followed by 40 cycles of denaturation at 95 °C for 15 s, annealing at 60 °C for 30 s, and extension at 72 °C for 20 s. Relative expression levels were evaluated using the 2^−∆∆Ct^ method [101]. Primers are listed in Additional file 2: Table S13.

### Virus-induced gene silencing in cotton

For the virus-induced gene silencing (VIGS) assays, approximately 500-bp fragments from the 13 selected secretome members were amplified from *G. hirsutum* cv. Zhongzhimian No.2 genomic DNA with previously designed primers [102]. Fragments were separately integrated into the pTRV2 vector and introduced into *A. tumefaciens* GV3101. *Agrobacterium* strains harboring the recombinant plasmid were combined with strains harboring the pTRV1 vector in a 1:1 ratio and co-infiltrated into cotyledons of 2-week-old *G. hirsutum* cv. Zhongzhimian No.2 seedling. The efficiency of the VIGS assay was evaluated using the essential for chloroplast development gene *cloroplastos alterados 1* (*CLA1*) as a control. The silencing efficiency of selected genes was determined by RT-qPCR, which compared gene expression in treated plants with gene expression in untreated plants collected at the same time, and with primers specific to the cotton *GhUbiquitin* gene as controls. Primers are listed in Additional file 2: Table S13.

Approximately 14 days following co-infiltration, white leaves were observed in plants in which the *CLA1* gene had been silenced by VIGS, at which point all plants were inoculated with 5.0 mL of *V. dahliae* Vd991 conidial suspension (5 × 10^6^ conidia/mL) using the root-dip method as described above. For each gene, 12 plants were used in three replicates. Verticillium wilt symptoms were evaluated using an established disease index [103] at 3 weeks after inoculation. In detail, the disease severity scores from cotton seedlings were divided into five grades: 0 = healthy, 1 = one true leaf showing yellowing, 2 = two true leaves showing wilt symptoms, 3 = two true leaves fallen off, and 4 = whole plant dead [103]. Differences between the inoculated and non-inoculated treatment groups were considered significant in paired Student’s *t*-tests with *P* ≤ 0.05. The vascular discoloration in shoots was assessed visually at four weeks after inoculation.

### Histochemical test for lignin

Freehand cross-sections from the base of the stem of both inoculated and mock-treated cotton plants (ZZM2 and cv. Junmian No.1) were obtained at 14 days after treatment, and lignin histochemistry was examined using the Wiesner reagent [104]. The cross-sections of stem tissue were incubated in a phloroglucinol solution (2% in 95% ethanol) for 10 min, and treated with 18% HCl for 5 min. Lignin accumulation levels were observed under a Leica fluorescence microscope (DM2500, Leica, Wetzlar, Germany).

### Supplementary Information

**Additional file 1: Fig. S1.** Flow chart illustrating the steps in the prediction of secretome in the cotton cultivar Zhongzhimian No.2 genome. **Fig. S2.** Length distribution of the predicted secreted proteins in cotton cultivar Zhongzhimian No.2. **Fig. S3.** Secretory characteristics of predicted proteins from cotton cultivar Zhongzhimian No.2. **Fig. S4.** Predictions of proteins with transmembrane domain within the genome of cotton cultivar Zhongzhimian No.2. **Fig. S5.** Statistics on the number of secreted proteins in the Zhongzhimian No.2 genome. **Fig. S6.** Comparison of the protein property of secretome versus the total encoded proteins of the Zhongzhimian No.2 genome. **Fig. S7.** Sequence alignment of 40 predicted secreted proteins from Zhongzhimian No.2 that cluster in main orthologue groups. **Fig. S8.** Synteny analysis of the coding regions of predicted secreted proteins from cotton cultivar Zhongzhimian No.2with each of the other 25 chromosomes. **Fig. S9.** Matrix representing the gene number of each chromosome and relationships between orthologues on the 26 chromosomes of Zhongzhimian No.2. **Fig. S10.** Expression of predicted secretome members in resistant and susceptible cotton cultivars in an infection time-course with *Verticillium dahliae*. **Fig. S11.** Gene expression pattern of predicted secretome members from cotton cultivar Zhongzhimian No.2 from three gene ontologyitems in response to *Verticillium dahliae*. **Fig. S12.** Histochemical analysis of lignin in stem cross-sections of resistance cultivar ZZM2 susceptible cultivar Junmian No.1 inoculated with *V. dahliae*. **Fig. S13.** GO enrichment of predicted secretome members in the resistant versus susceptible cotton cultivar in response to *Verticillium dahliae*. **Fig. S14.** Flow chart of representative members from the secretome of allotetraploid cotton cultivar Zhongzhimian No.2. **Fig. S15.** Sequence alignment the members of GhSec137 orthologue groups.

**Additional file 2: Table S1.** List of the predicted secreted proteins in the cotton cultivar Zhongzhimian No.2 genome. **Table S2.** Statistical analysis of the orthologue relationship among the cultivar Zhongzhimian No.2 predicted secretome. **Table S3.** Statistics of the gene numbers of the predicted secretome and genome with functional annotation. **Table S4.** InterPro annotation of the predicted secretome of cotton cultivar Zhongzhimian No.2. **Table S5.** Gene function network annotation of secretome by KEGG database. **Table S6.** Information on the predicted secretome members in resistant and susceptible cultivars in response to *Verticillium dahliae*. **Table S7.** GO enrichment of the predicted secretome members in the cotton response to *Verticillium dahliae*. **Table S8.** Gene expression pattern of predicted secretome members indicates involvement of the phenylpropanoid biosynthesis pathway. **Table S9.** List of genes associated with extracellular stress that were only induced in the resistant but not the susceptible cotton cultivar during *Verticillium dahliae* infection. **Table S10.** Gene expression patterns of predicted secretome members that were co-expressed in the resistant or susceptible cultivars of cotton in response to *Verticillium dahliae*. **Table S11.** Statistic of members of orthologue associated with the 13 Verticillium wilt resistance candidate genes. **Table S12.** Orthologue analysis of 13 Verticillium wilt resistance candidate genes among other three cotton genomes. **Table S13.** Primer used in this study.

## Data Availability

This Whole Genome Shotgun project of *Gossypium hirsutum* cultivar Zhongzhimian No.2 has been deposited at DDBJ/ENA/GenBank under the accession JAMQUR000000000 [105]. The version described in this paper is version JAMQUR010000000. The BioProject accession is PRJNA846595 [106]. The RNA-seq data (resistant cultivar *G. hirsutum* Zhongzhimian No.2 and susceptible cultivar *G. hirsutum* Jianmian No.1 responses to *Verticillium dahliae*) presented in this study have been deposited in the NCBI Sequence Read Archive (SRA) database under project accession number PRJNA844504 [107].
