# Supplementary material for: Genome-wide identification and analysis of a cotton secretome reveals its role in resistance against Verticillium dahliae
Source: BMC Biol. 2023 Aug 4;21:166. doi: 10.1186/s12915-023-01650-x (PMC10403859; doi:10.1186/s12915-023-01650-x)
Supplement: Supplementary file 1 — Additional file 1: Fig. S1. Flow chart illustrating the steps in the prediction of secretome in the cotton cultivar Zhongzhimian No.2 genome. Fig. S2. Length distribution of the predicted secreted proteins in cotton cultivar Zhongzhimian No.2. Fig. S3. Secretory characteristics of predicted proteins from cotton cultivar Zhongzhimian No.2. Fig. S4. Predictions of proteins with transmembrane domain within the genome of cotton cultivar Zhongzhimian No.2. Fig. S5. Statistics on the number of secreted proteins in the Zhongzhimian No.2 genome. Fig. S6. Comparison of the protein property of secretome versus the total encoded proteins of the Zhongzhimian No.2 genome. Fig. S7. Sequence alignment of 40 predicted secreted proteins from Zhongzhimian No.2 that cluster in main orthologue groups. Fig. S8. Synteny analysis of the coding regions of predicted secreted proteins from cotton cultivar Zhongzhimian No.2with each of the other 25 chromosomes. Fig. S9. Matrix representing the gene number of each chromosome and relationships between orthologues on the 26 chromosomes of Zhongzhimian No.2. Fig. S10. Expression of predicted secretome members in resistant and susceptible cotton cultivars in an infection time-course with Verticillium dahliae. Fig. S11. Gene expression pattern of predicted secretome members from cotton cultivar Zhongzhimian No.2 from three gene ontologyitems in response to Verticillium dahliae. Fig. S12. Histochemical analysis of lignin in stem cross-sections of resistance cultivar ZZM2 susceptible cultivar Junmian No.1 inoculated with V. dahliae. Fig. S13. GO enrichment of predicted secretome members in the resistant versus susceptible cotton cultivar in response to Verticillium dahliae. Fig. S14. Flow chart of representative members from the secretome of allotetraploid cotton cultivar Zhongzhimian No.2. Fig. S15. Sequence alignment the members of GhSec137 orthologue groups. [file 12915_2023_1650_MOESM1_ESM.pdf]

Fig. S1

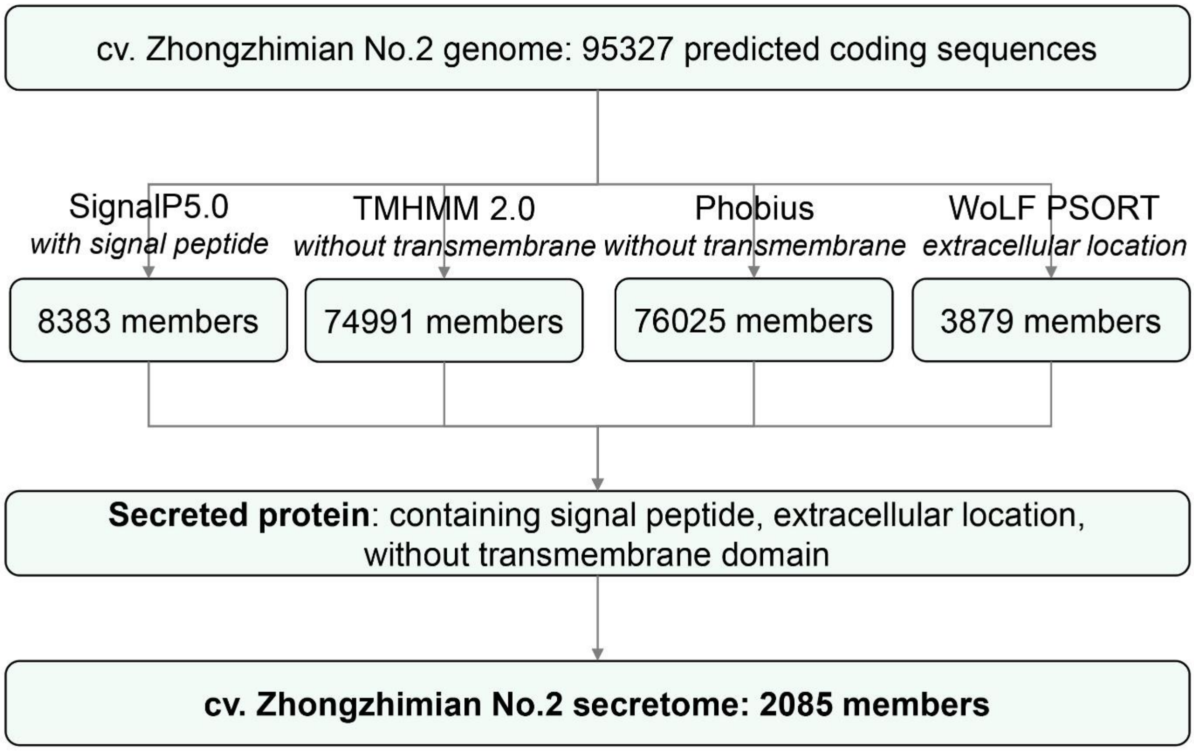

Fig. S1 Flow chart illustrating the steps in the prediction of secretome in the cotton cultivar Zhongzhimian No.2 genome. Secreted proteins were defined as those having a signal peptide (SP), lacking transmembrane (TM) domains, and of extracellular location. The subcellular location of the encoded proteins was predicted using the plant-model of WolfPsort.

Fig. S2

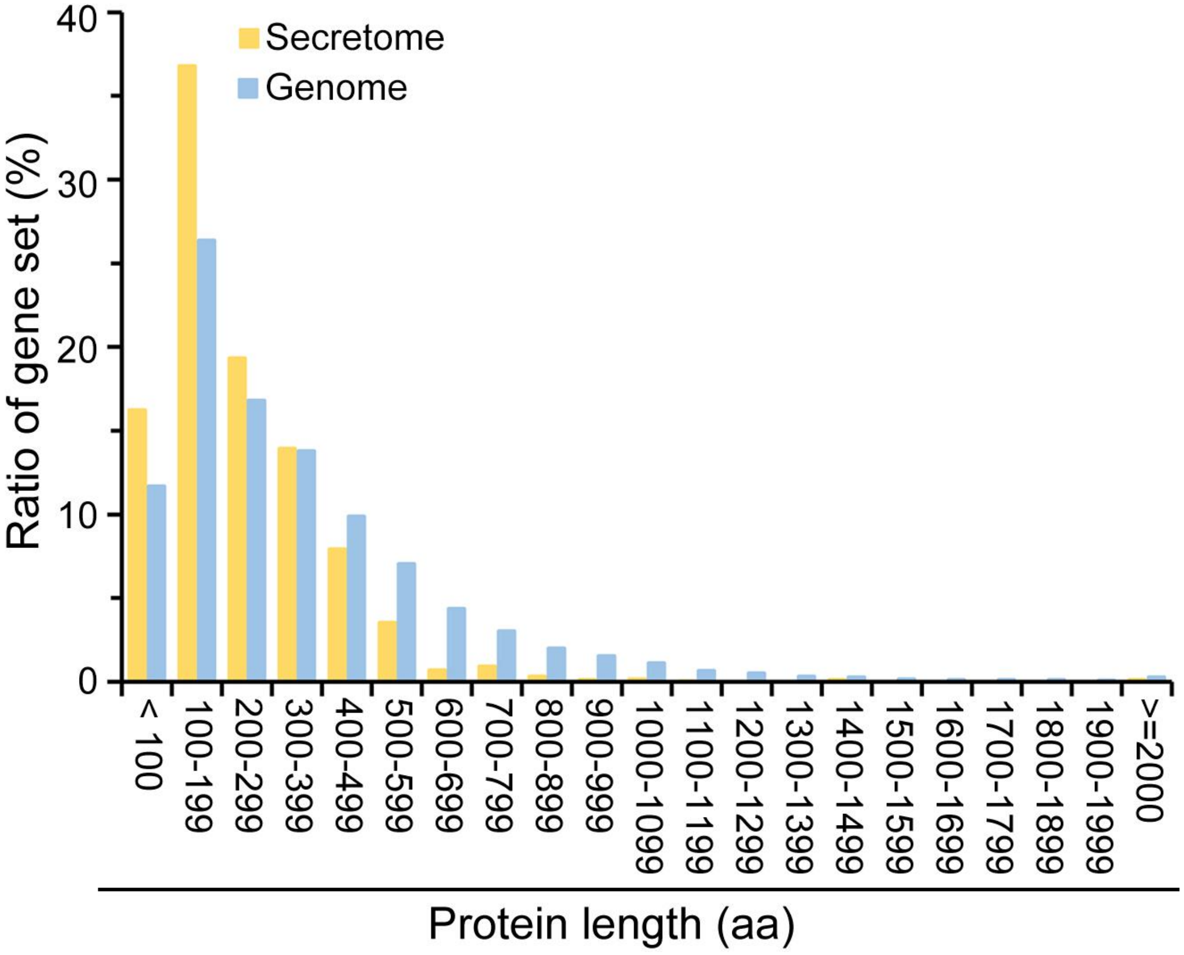

Fig. S2 Length distribution of the predicted secreted proteins in cotton cultivar Zhongzhimian No.2. The distribution was calculated for each gene set relative to the total secreted proteins. The total predicted proteins of the genome were used for the comparison group.

Fig. S3

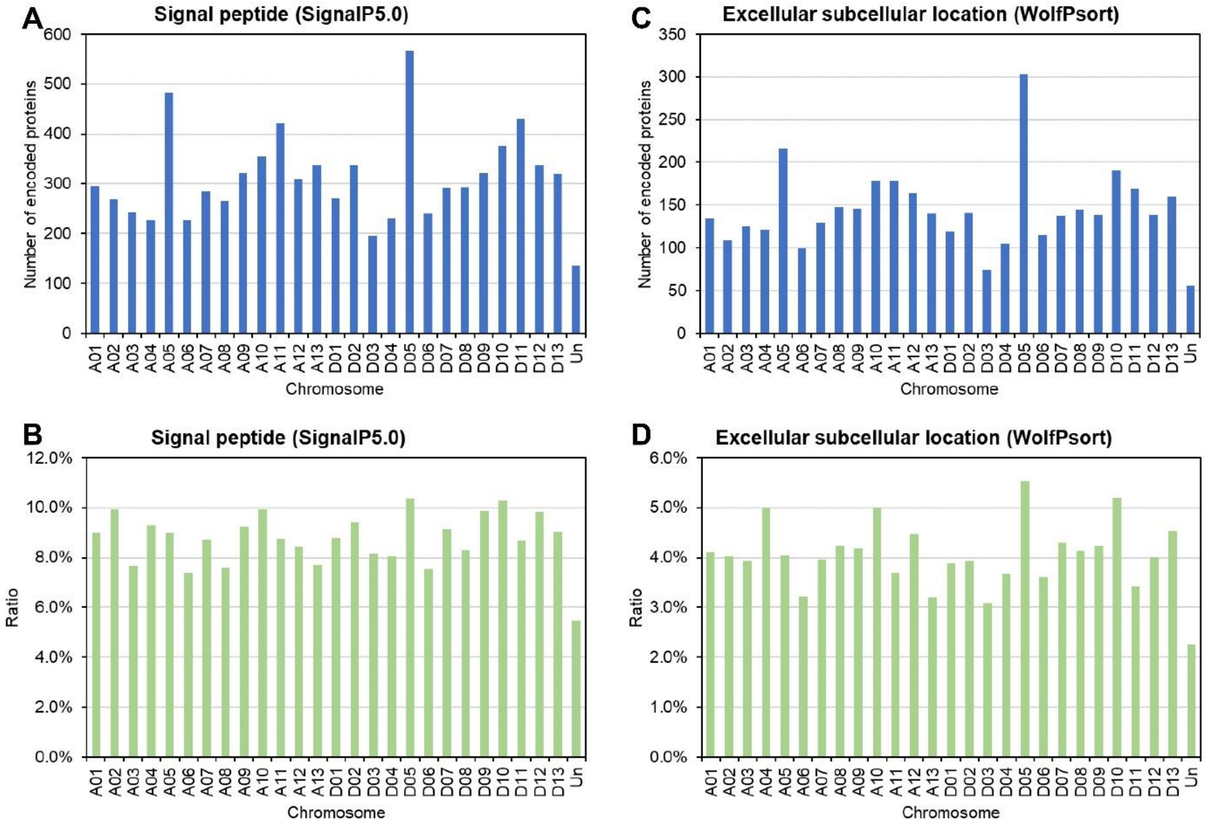

Fig. S3 Secretory characteristics of predicted proteins from cotton cultivar Zhongzhimian No.2. **A**, **B** Encoded proteins with signal peptide. **A** Number of encoded proteins with signal peptide in 26 chromosomes. **B** Ratio of encoded proteins with signal peptide in each chromosome relative to the total encoded proteins in the relative chromosomes. **C**, **D** The subcellular location of encoded proteins. **C** Number of encoded proteins with an extracellular location from the 26 chromosomes. **D** Ratio of encoded proteins with an extracellular location in each chromosome relative to the total encoded proteins in the relative chromosomes.

Fig. S4

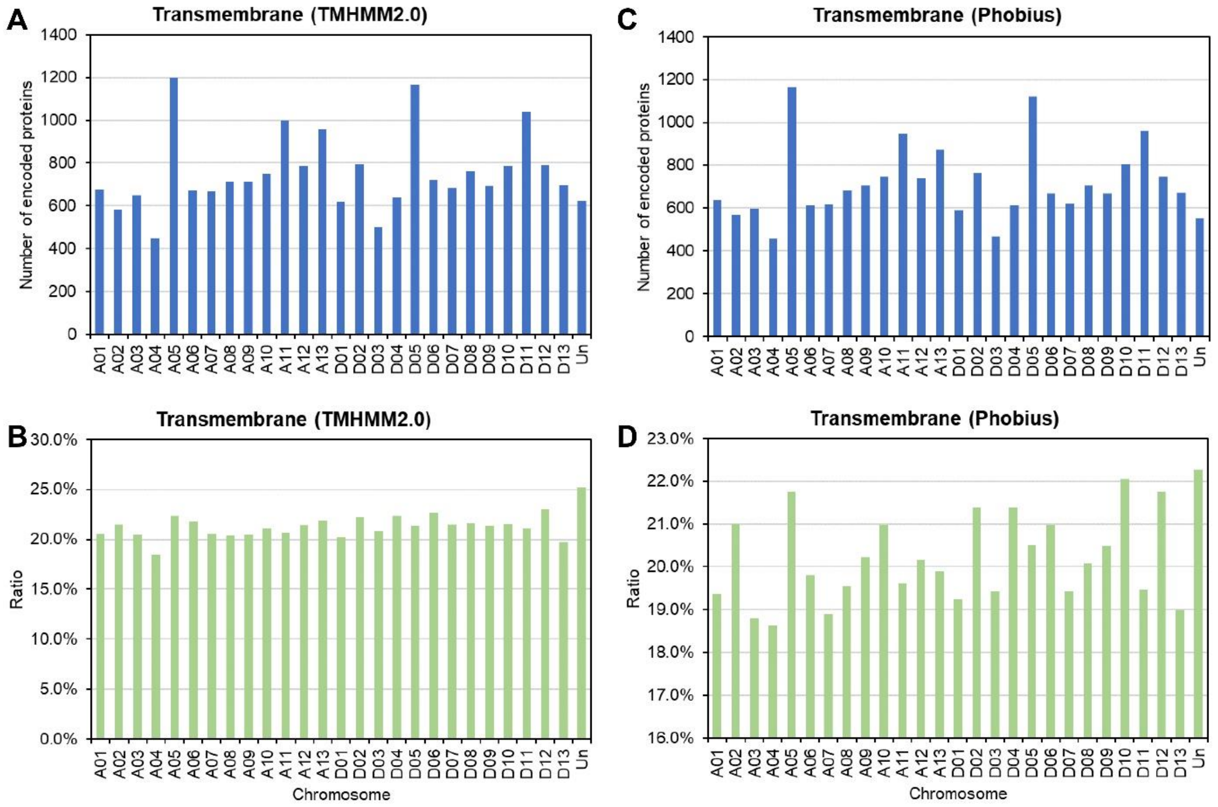

Fig. S4 Predictions of proteins with transmembrane domain within the genome of cotton cultivar Zhongzhimian No.2. The transmembrane domain of encoded proteins predicted by TMHMM2.0 **A**, **B** and Phobius **C**, **D**. **A, C** Number of encoded proteins with a transmembrane domain in 26 chromosomes. **B, D** Ratio of encoded proteins with a transmembrane domain on each chromosome versus the total encoded proteins in the relative chromosomes.

Fig. S5

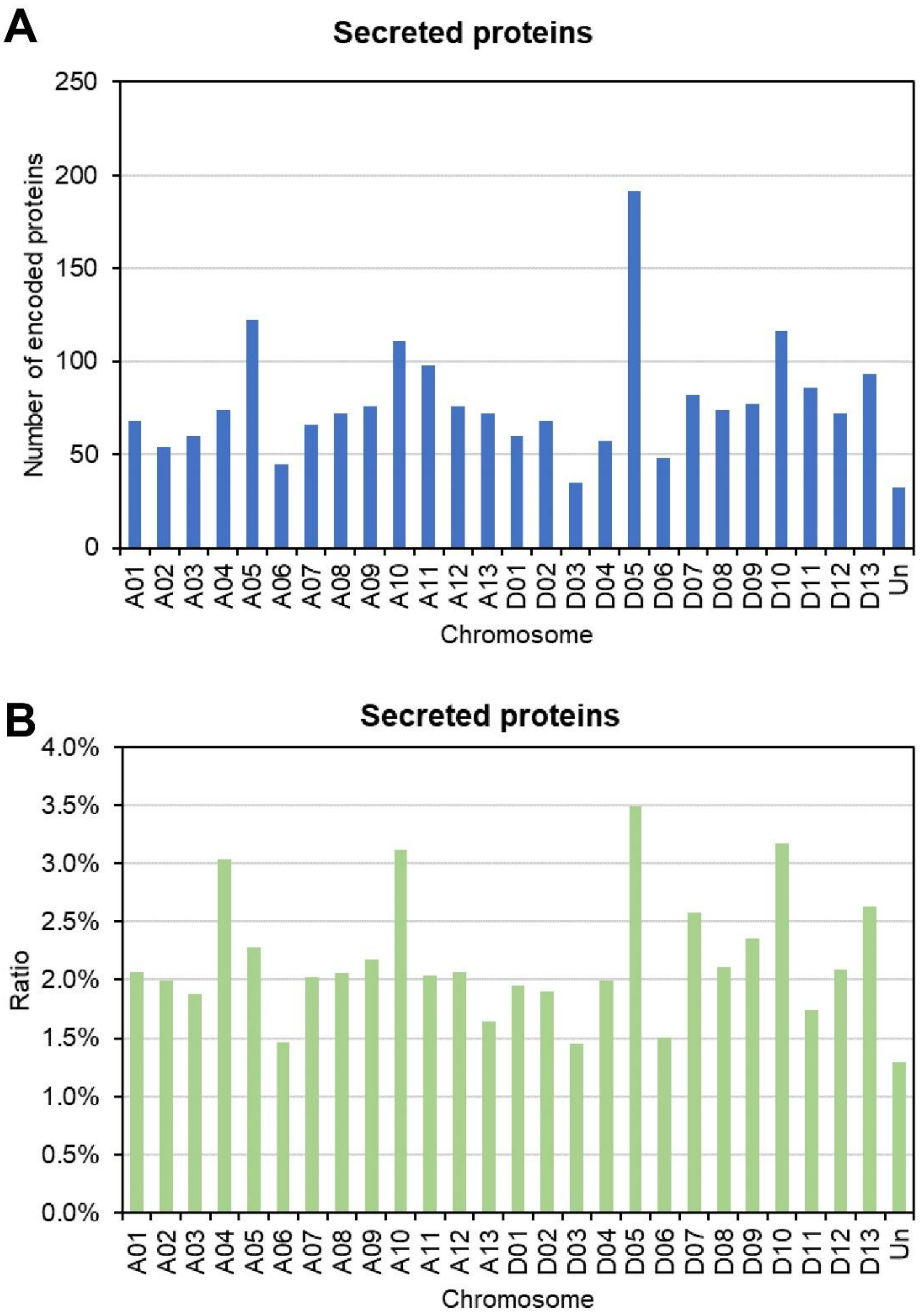

Fig. S5 Statistics on the number of secreted proteins in the Zhongzhimian No.2 genome. **A** Number of predicted secreted proteins encoded in 26 chromosomes. **B** Ratio of secreted proteins in each chromosome versus the total encoded proteins in the relative chromosomes.

Fig. S6

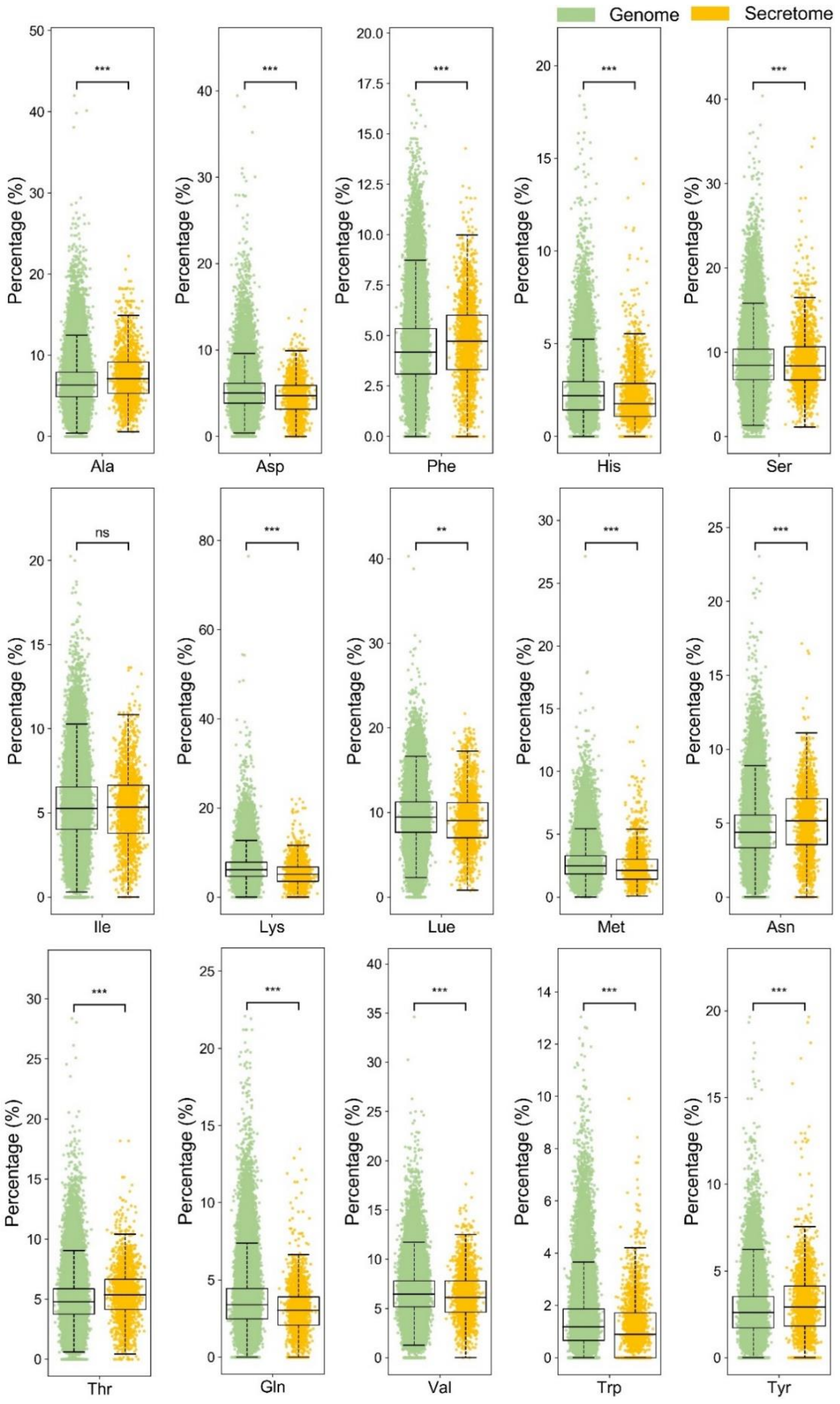

Fig. S6 Comparison of the protein property of secretome versus the total encoded proteins of the Zhongzhimian No.2 genome. The length ratio of CDS/gene represents the value of coding

sequence length compared to the gene length; the value of gene without intron is 1.0. Asterisks (***) represent statistical significance at *P* < 0.001 based on unpaired Student's *t*-tests, and Levene's test was used to assess variance homogeneity.

Fig. S7

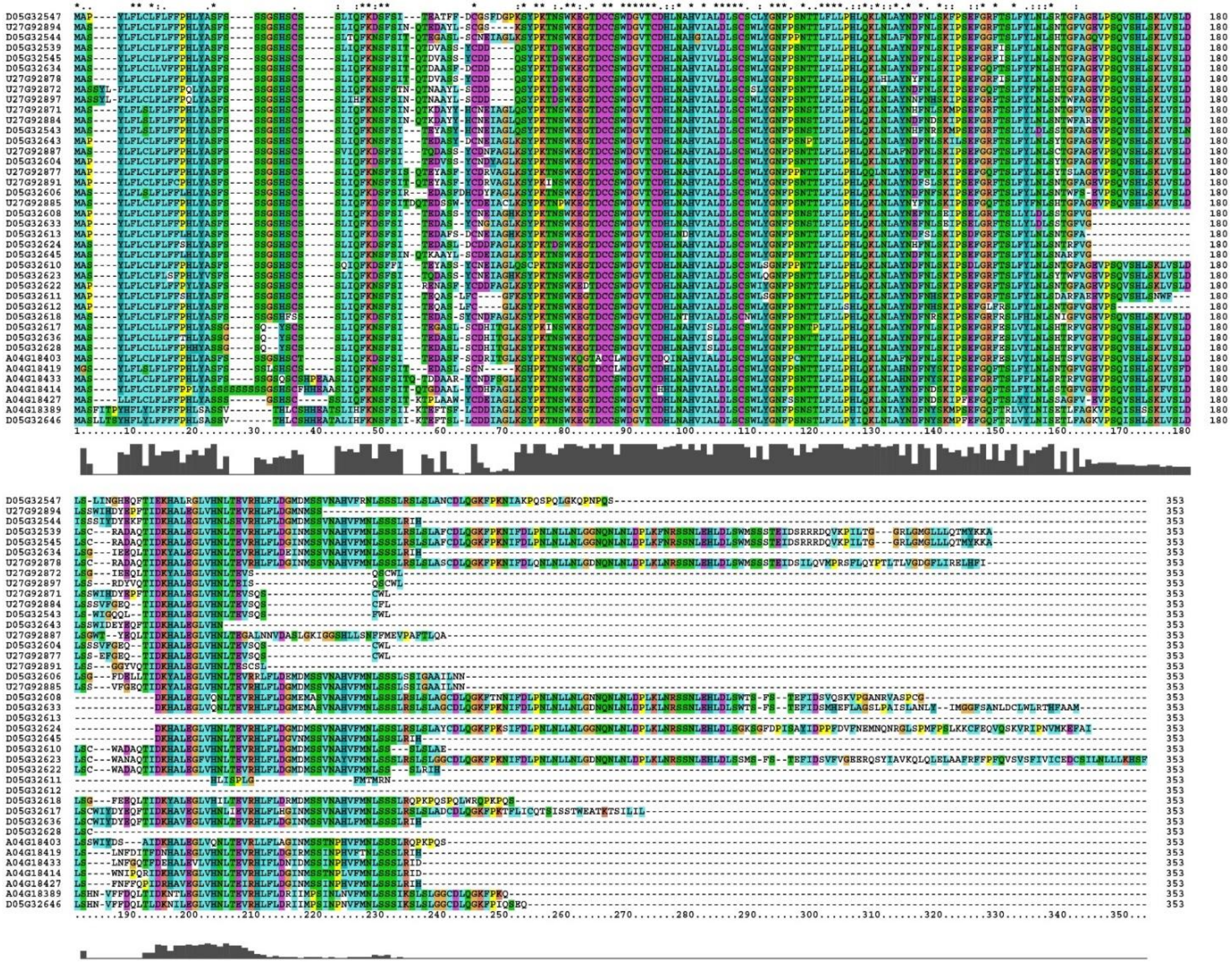

Fig. S7 Sequence alignment of 40 predicted secreted proteins from Zhongzhimian No.2 that cluster in main orthologue groups. Sequence alignment

was performed with using Clustal X2. Asterisks represent the absolutely conserved sites, and the dot (.) and (:) represents the variable sites with different or similar residue properties compared the consensus residue, respectively.

Fig. S8

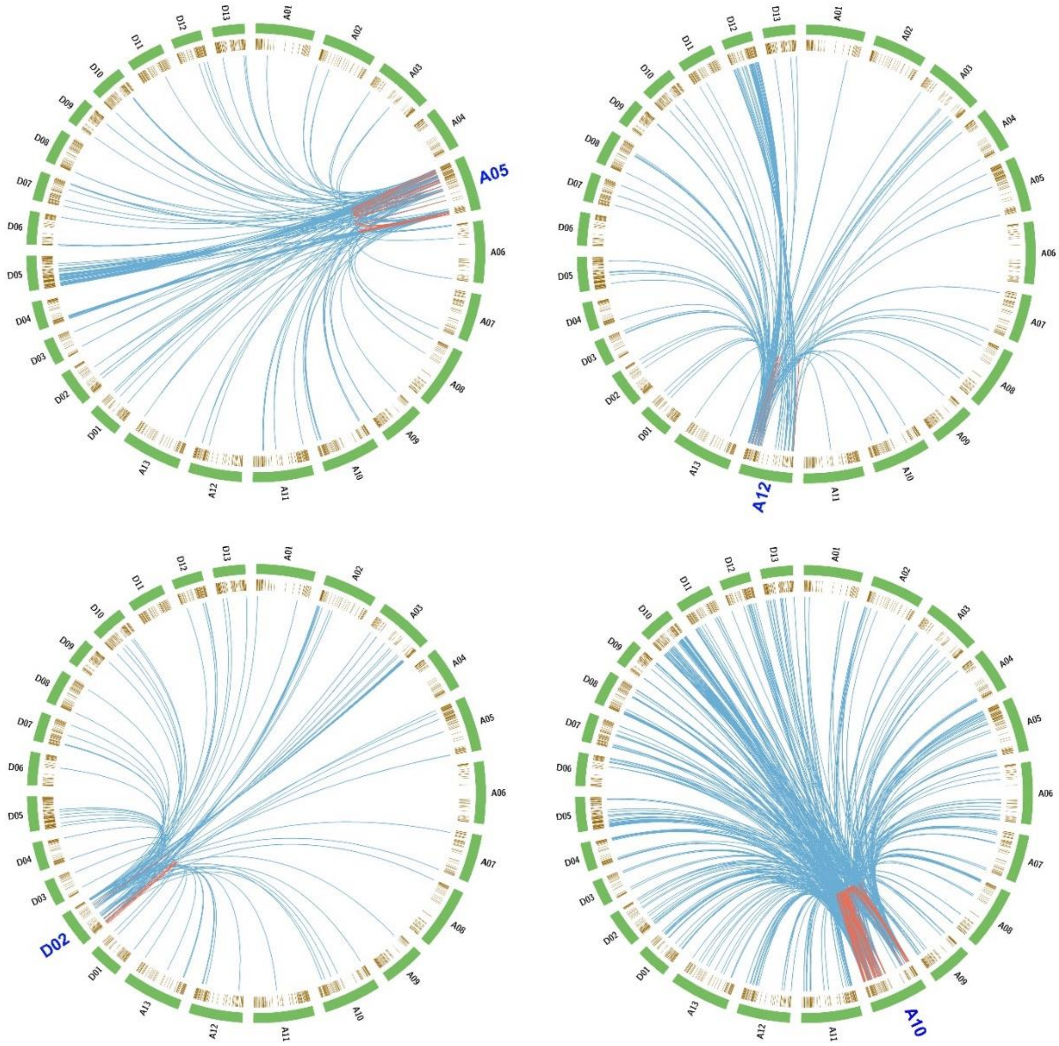

Fig. S8 Synteny analysis of the coding regions of predicted secreted proteins from cotton cultivar Zhongzhimian No.2 (ZZM2) with each of the other 25 chromosomes. The synteny relationship was constructed by orthologue clustering (both coverage and identities up to 30%) of predicted secreted proteins from A05, A10, A10, or D02 chromosomes with other predicted secreted proteins from 25 chromosomes, present in blue lines; and red lines represents the self-orthologs of secreted proteins within chromosome. Outer circle with green blocks represents 26 chromosomes of ZZM2 genome, and inner circle with brown lines represents the secreted proteins encode by ZZM2 genome.

Fig. S9

A

Chromosomes

	A01	A02	A03	A04	A05	A06	A07	A08	A09	A10	A11	A12	A13	D01	D02	D03	D04	D05	D06	D07	D08	D09	D10	D11	D12	D13
A01	26	1	6	6	2	1	1	3	1	3	2	3	3	38	3	1	5	5	1	1	2	2	2	1	5	4
A02	1	11	5	2	3	4	2	5	1	6	8	2	3	1	15	11	1	3	5	2	5	3	6	7	3	4
A03	5	2	11	2	8	2	6	1	3	6	4	17	9	7	25	15	5	8	2	8	3	3	5	3	12	6
A04	9	9	2	35	5	3	1	6	0	2	2	3	2	16	2	2	16	32	3	9	3	10	3	10	3	4
A05	8	3	17	5	45	10	3	8	7	15	16	13	9	12	15	8	26	73	9	10	11	4	11	14	17	11
A06	4	6	3	6	9	14	1	8	3	9	11	3	4	5	6	3	4	8	28	1	7	0	11	8	4	5
A07	2	2	5	1	3	1	16	5	4	3	7	7	3	2	3	2	0	0	1	37	5	3	2	7	6	5
A08	3	5	3	4	7	7	3	24	3	9	8	8	5	4	4	3	2	14	6	4	45	2	7	8	7	5
A09	1	4	2	0	8	2	3	6	34	14	2	3	0	2	4	0	3	2	1	5	5	43	10	0	4	0
A10	5	6	8	2	10	6	3	9	10	55	9	10	5	7	9	5	6	10	7	9	11	7	78	8	7	5
A11	3	9	7	3	12	9	15	9	2	10	36	3	9	3	5	8	2	10	9	18	11	1	10	66	6	19
A12	4	2	16	4	7	2	7	7	4	12	5	13	9	6	8	8	1	6	3	7	10	4	11	6	47	13
A13	3	1	8	2	6	2	5	4	0	4	6	6	22	2	7	3	4	4	1	3	6	1	3	6	7	53
D01	34	1	6	6	5	3	1	6	3	7	3	5	2	11	5	2	6	7	1	4	2	1	3	1	7	2
D02	3	17	23	2	7	4	2	4	3	10	4	5	9	5	15	4	4	7	6	4	5	4	8	4	8	7
D03	1	12	16	2	7	3	2	2	0	6	7	9	5	3	4	14	2	10	4	5	5	0	4	7	5	6
D04	10	1	10	16	20	1	0	2	2	5	1	1	4	11	10	1	13	11	2	3	1	2	5	1	7	5
D05	9	2	11	60	91	9	0	15	3	12	28	20	15	36	9	11	14	123	10	6	11	1	10	28	19	17
D06	2	4	2	4	7	24	1	4	2	6	8	4	1	2	4	3	3	8	9	0	5	0	7	6	3	1
D07	2	7	6	2	7	1	46	5	7	7	11	7	2	4	5	3	3	4	0	29	6	6	4	12	7	4
D08	5	7	5	6	9	9	3	45	2	12	11	10	6	5	7	5	3	12	10	5	24	3	9	12	9	8
D09	2	4	2	3	4	0	3	3	44	14	1	4	1	1	5	0	2	1	0	5	3	29	9	1	3	2
D10	2	8	5	3	6	7	1	8	8	90	8	8	4	3	7	3	5	7	6	3	8	8	55	6	6	6
D11	2	7	6	4	10	6	16	8	0	9	66	4	8	2	4	8	2	10	7	22	12	1	7	41	5	19
D12	8	3	15	6	7	3	7	7	6	8	7	49	8	9	12	8	2	7	2	8	11	5	8	6	19	13
D13	5	2	5	4	10	3	5	5	0	4	11	9	62	2	5	4	6	7	1	4	9	2	5	9	10	34

5 15 Max.

B

Chromosomes

	A01	A02	A03	A04	A05	A06	A07	A08	A09	A10	A11	A12	A13	D01	D02	D03	D04	D05	D06	D07	D08	D09	D10	D11	D12	D13
A01	19	1	3	0	1	0	1	0	1	2	1	1	2	32	2	0	0	0	0	1	0	0	2	0	2	3
A02	1	6	0	0	2	0	2	2	0	3	5	0	0	1	11	9	0	2	0	1	2	1	3	2	0	1
A03	3	0	9	1	2	0	2	0	1	3	0	8	6	3	23	14	3	3	0	3	0	0	3	0	5	4
A04	0	0	1	31	0	1	0	0	0	0	0	2	1	0	0	1	13	26	0	0	1	1	0	0	2	2
A05	1	3	3	0	39	5	1	4	3	11	6	9	4	5	7	6	18	59	4	7	3	2	9	1	8	5
A06	0	0	0	1	5	7	0	2	1	3	1	0	0	1	0	0	0	5	26	1	1	0	3	0	1	0
A07	2	2	3	0	1	0	11	2	2	2	5	2	2	2	2	1	0	0	0	35	1	0	2	5	2	3
A08	0	3	0	0	4	3	2	17	1	5	5	5	2	2	3	3	0	3	2	3	37	0	3	3	5	2
A09	1	0	1	0	3	1	1	1	27	5	1	1	0	2	1	0	1	0	0	2	0	40	2	0	2	0
A10	2	2	2	0	6	3	1	4	4	37	2	4	1	6	5	2	2	4	3	4	1	1	71	0	4	1
A11	1	5	0	0	4	1	13	4	1	2	24	1	4	1	1	4	0	2	0	15	4	0	3	57	3	14
A12	2	0	7	3	2	0	2	4	2	5	1	7	2	4	4	4	1	4	0	3	5	2	5	2	44	4
A13	2	0	5	1	2	0	3	3	0	1	4	2	13	1	4	1	3	2	0	3	5	0	1	4	1	46
D01	31	1	2	0	4	2	1	3	3	6	2	3	1	9	3	1	1	1	0	4	0	0	3	0	5	2
D02	2	12	19	0	3	0	1	1	1	5	1	3	4	3	11	2	2	3	2	3	2	1	5	0	3	3
D03	0	8	14	1	3	0	1	1	0	3	3	4	2	2	3	9	2	5	0	2	2	0	3	2	2	3
D04	0	0	2	13	17	0	0	0	1	2	0	1	3	1	2	1	13	1	0	1	0	2	2	0	0	2
D05	0	2	4	50	78	5	0	2	0	7	11	16	12	3	6	8	3	108	5	5	4	0	6	9	13	14
D06	0	0	0	0	4	24	0	1	0	3	0	0	0	0	1	0	0	5	4	0	1	0	3	0	0	0
D07	2	4	3	0	5	1	39	3	3	5	7	3	2	4	4	1	1	3	0	18	1	0	3	6	4	4
D08	0	3	0	1	3	2	1	38	0	3	5	5	3	0	4	4	0	4	2	1	13	0	3	4	4	4
D09	0	1	0	1	2	0	0	0	39	1	0	2	0	0	1	0	2	0	0	0	0	18	1	0	1	0
D10	2	2	2	0	4	3	1	1	2	86	3	4	1	3	5	2	2	3	3	2	1	1	46	1	4	1
D11	0	3	0	0	1	0	15	4	0	0	59	2	4	0	0	3	0	1	0	16	5	0	1	31	3	15
D12	3	0	6	3	3	1	2	4	3	5	3	44	1	5	4	4	0	3	0	4	4	1	5	3	8	3
D13	3	1	3	2	4	0	3	4	0	1	7	3	56	2	3	2	3	4	0	4	7	0	1	6	2	29

5 10 Max.

Fig. S9 Matrix representing the gene number of each chromosome and relationships between orthologs on the 26 chromosomes of Zhongzhimian No.2. The data in columns represents the gene number of each chromosome (top labels) in orthologue clustering with other chromosomes (left

labels). **A** Orthologue clustering by coverage and identities up to 50% and **B** orthologue clustering by coverage and identities up to 70%.

Fig. S10

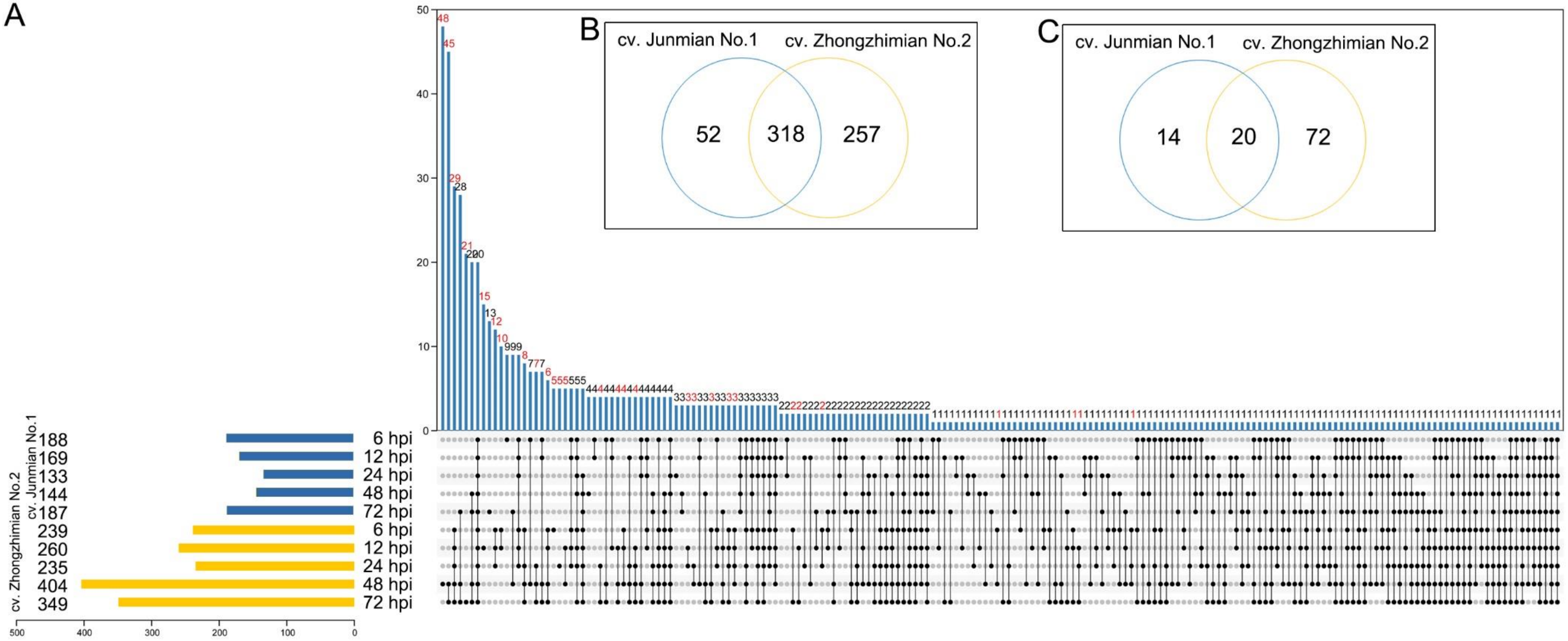

Fig. S10 Expression of predicted secretome members in resistant and susceptible cotton cultivars in an infection time-course with *Verticillium dahliae*. Cultivar Zhongzhimian No.2 and cv. Junmian No.1 are the resistant and susceptible cultivars, respectively. The infection time-course including 6, 12, 24, 48, 72 hours post-inoculation points. Filtered parameters of DEGs is $|\log_2 FoldChange| \geq 1.0$ and *Padj* < 0.05. **A** Venn diagram representing the relationship of DEGs at each infection time point of the susceptible and resistant cultivar in response to the *V. dahliae*. The number

in red color represents the DEGs responsive in the resistant cultivar but not responsive in the susceptible cultivar. **B** Venn diagram to present the total DEGs of the resistant and susceptible cultivar in response to *V. dahliae*. **C** The Venn diagram represents the DEGs co-expressed at all time points between resistant and susceptible cultivars in response to *V. dahliae*.

Fig. S11

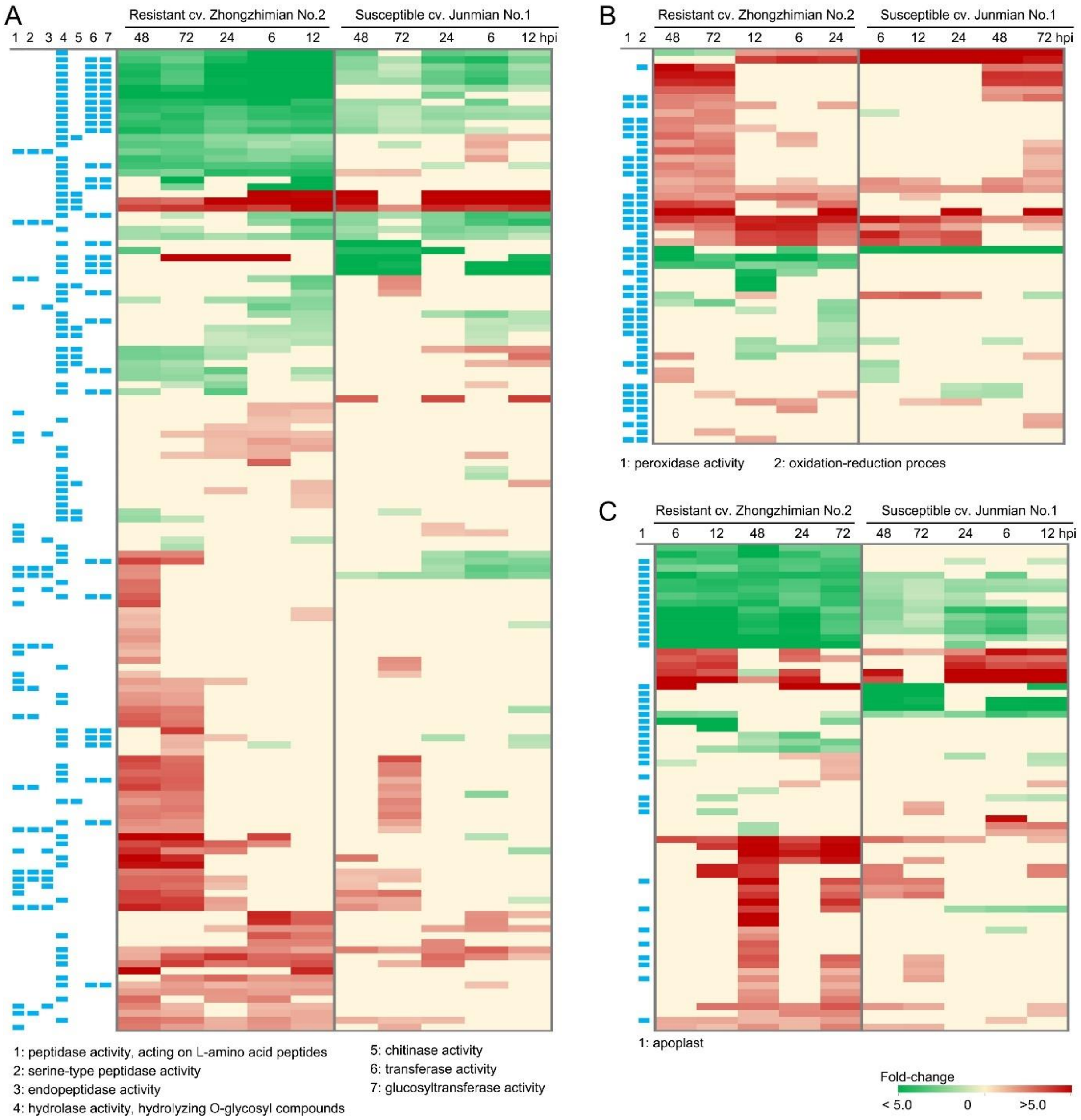

Fig. S11 Gene expression pattern of predicted secretome members from cotton cultivar Zhongzhimian No.2 from three gene ontology (GO) items in response to *Verticillium dahliae*. **A** GO:0016787, hydrolase activity. **B** GO:0055114, oxidation-reduction process. **C** GO:0005576, extracellular region.

Fig. S12

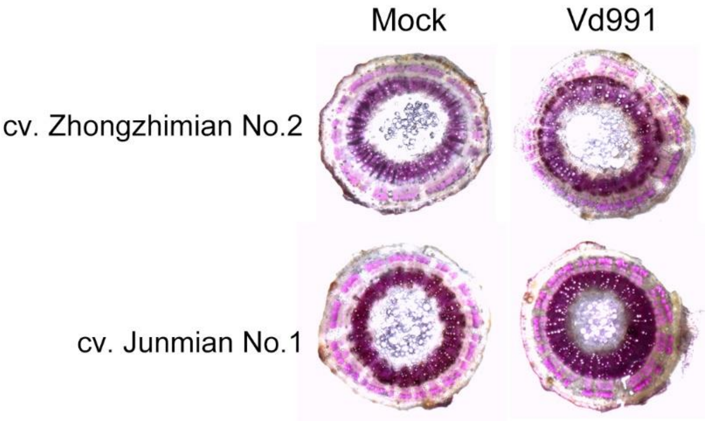

Fig. S12 Histochemical analysis of lignin in stem cross-sections of resistance cultivar ZZM2 susceptible cultivar Junmian No.1 inoculated with *V. dahliae*. Hand-cut cross-sections of stem were stained with Wiesner reagents for detecting lignin, sections from inoculated plants 14 d after treatment. Pink staining with the Wiesner reagent indicates the presence of p-hydroxycinnamyl aldehyde end groups in lignin.

Fig. S13

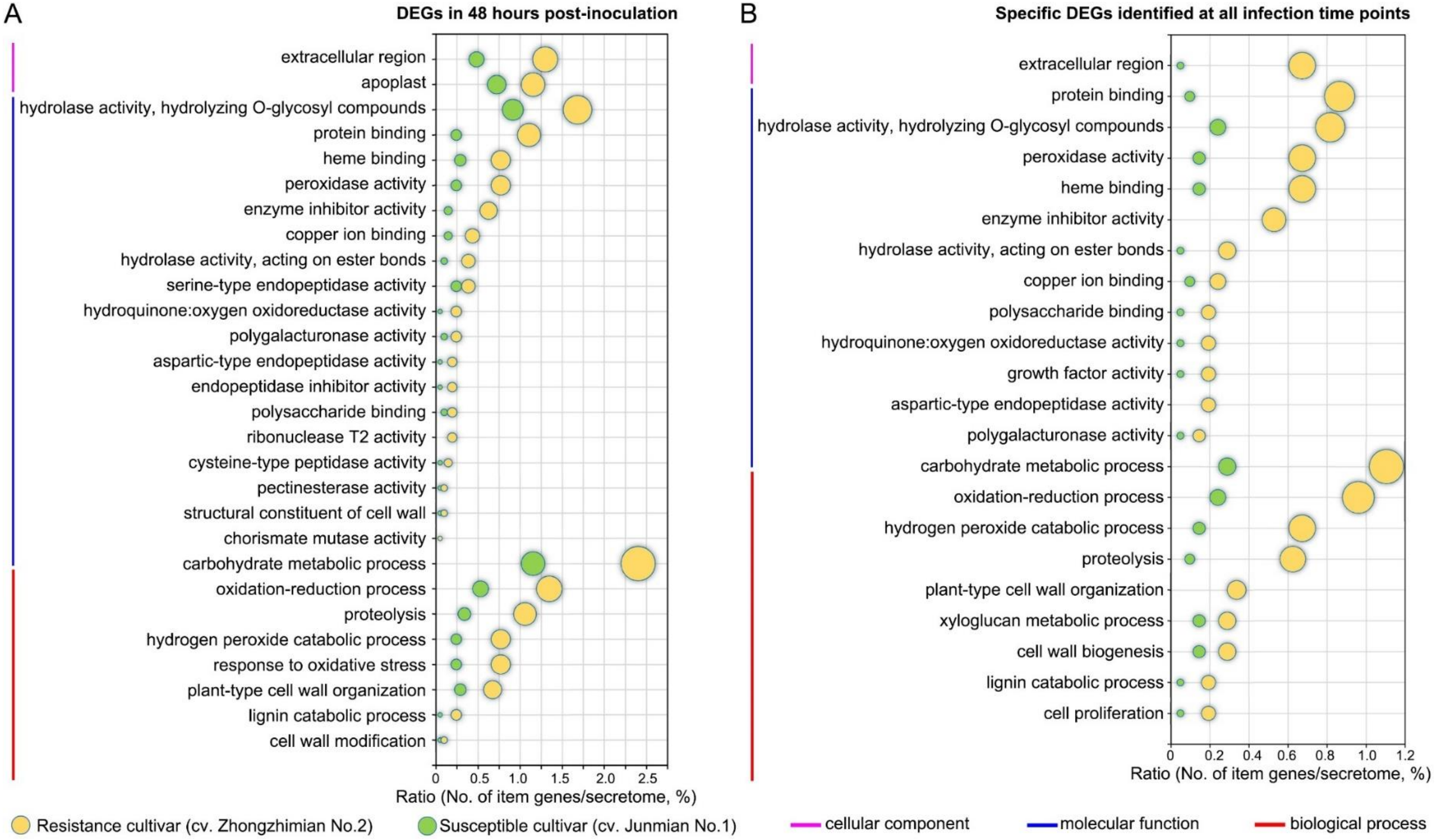

Fig. S13 GO enrichment of predicted secretome members in the resistant versus susceptible cotton cultivar in response to *Verticillium dahliae*. **A** Enrichment of DEGs in the resistant versus susceptible cultivar at 48 hours post-inoculation. Cultivar Zhongzhimian No.2 and cv. Junmian No.1 are

the resistant and susceptible cultivars, respectively. **B** Enrichment of DEGs induced in resistant versus susceptible cultivars during all infection time points.

Fig. S14

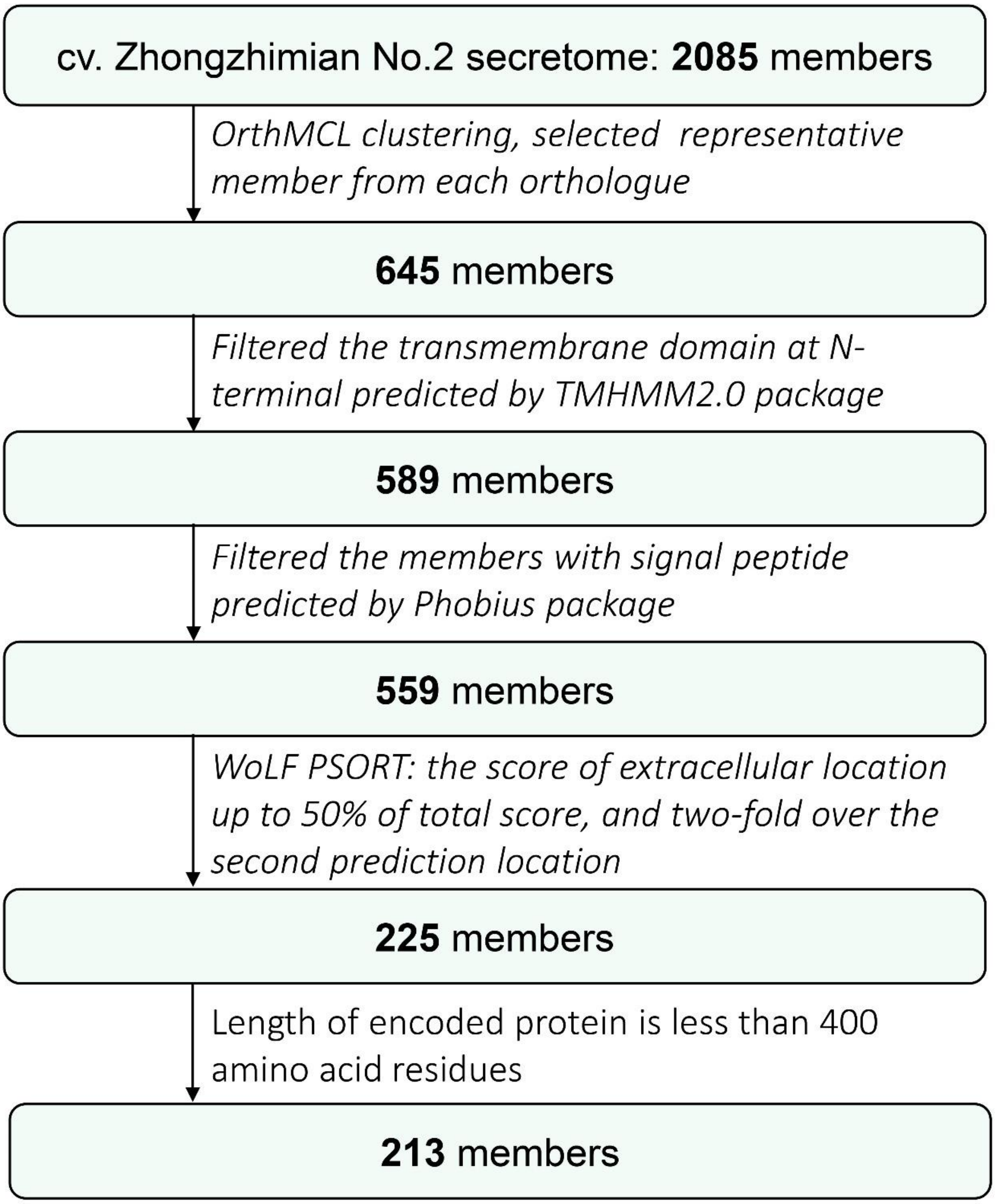

Fig. S14 Flow chart of representative members from the secretome of allotetraploid cotton cultivar Zhongzhimian No.2 (ZZM2). The representative members were selected from each gene-family from the clustering analysis with OrthMCL.

Fig. S15

```
            *          : : :      .           ::::  *::: *: **  :*:*:  *
A05G30268   MKN-----LFVIIFAFLVCQANAFRPILLLGN-QNLIERTCKQTPFYDLCIWSLKSNPES    54
D05G29550   MKN-----LIVIIFAFLVCQANAFRPILLLGN-QNLIERTCKQTPFYDLCIWSLKSNPES    54
A10G67021   MNNTVSFALLHFAFCFTLVSFTPISALELHGTGANMVETTCQKTPFYNLCVSALKSDPRS    60
D10G64907   MNNTISLTLLHIAFCFTLVSFTPISALELHGTGANMVETTCQKTPFYNLCVSALKSDPRS    60
D01G00433   MNT----HSLKLLLILLTITFPST------QCDDDLVDQICKKTPFYDLCISTLKSN--S    48
A01G00592   MPQN---LKAPLLFSLILGFLATQAISISNKKTLDLIEQTCRQSGFFALCDSTLRSDPQS    57
D01G00434   MPQN---LKAPLLFSLILGFLATQAISISNKKTMDLIEQTCRQSGFFALCNSTLRSDPRS    57
            1.......10........20........30........40........50........60

             . .:  **.: .: :  :*. **  *   ::   . ** :  :   * : *   :.  :
A05G30268   RDASVKKLAQIMVDSLKTKATETLDLIDELLQDGLALDPEMQKALTSCAERYNVIIRGDV    114
D05G29550   RDASVKKLAQIMVDSLKTKATETLDLIDELLQDGLALDPEMQKALTSCAERYNVIIRGDV    114
A10G67021   SGANVAGLAQIGTDKLKAKATATLRQITALLK--VAKDPKLKMALRDCADYYNAIVKYDI    118
D10G64907   SGADVAGLAQIGTDKLKAKATATLRQITALLK--VAKDPKLKMALRDCADYYNAIVKYDI    118
D01G00433   NGKDVKGLASVMADTMLSNATDTLSYIRAEIN--RTPDPKIERALAYCAELYIPVVKYNL    106
A01G00592   SNAKLEGLAKISVEIVIDKANATLNFIVDLFKN--VSDPVLYRSYGTCIDSYDASVERLL    115
D01G00434   SNANLEGLAKISVEIVIDKANATLNFIVDLFKN--VSDPVLYRSYGTCIDSYGASVQRLL    115
            ........70........80........90.......100.......110.......120

            *    *: . :   . ..   .* :.::*   :       .:   *:::*.:* : ..*:
A05G30268   PEINEALKTGDYKFAAKGANDAAIEANSCEIEFS-TKS--PLTDMNKVVHDVSVVAASIV    171
D05G29550   PEINEALKTGDYKFAAKGANDAAIEANSCEIEFP-TKS--PLTDMNKVVHDVSVVAASIV    171
A10G67021   PVAVEAVTKGDPKFGVEGATDAANEADACGRGFK-NQPRFPIYASNKVVHDLSAVVASIV    177
D10G64907   PVAVEAVTKGDPKFGVEGATDAANEADACGRGFK-NQPKFPIYASNKVVHDLSAVVASIV    177
D01G00433   PQAIDALSKGQFEFAADGISDAAKEADSCEKMISWSQELAALSDRNKLIHSLSDVAVAIV    166
A01G00592   PKAIAALSSKDYATSRHDAATVAINVNACDKQFS---EKTPFSDRNRLVHDLSLMSAGII    172
D01G00434   PEAIAALGSKDYATSRHDVATVATNVNACDEQFP---EKTPFSDRNRLVHDLSLMSAGII    172
            .......130.......140.......150.......160.......170.......180

            :::
A05G30268   KIIQTNQNAIKQIKACGNFPGATLSCLLPYQLIPAMENWVLKAVPREANNATGSLAKAAL    231
D05G29550   KIIQTKFSF---------------------------------------------------    180
A10G67021   QLLL--------------------------------------------------------    181
D10G64907   QLLL--------------------------------------------------------    181
D01G00433   KILLKG------------------------------------------------------    172
A01G00592   ELLG--------------------------------------------------------    176
D01G00434   ELLG--------------------------------------------------------    176
            .......190.......200.......210.......220.......230.......240

A05G30268   STSNHFLFMYDSGGEADSCLLMFVLFLF    259
D05G29550   ----------------------------    180
A10G67021   ----------------------------    181
D10G64907   ----------------------------    181
D01G00433   ----------------------------    172
A01G00592   ----------------------------    176
D01G00434   ----------------------------    176
            .......250.......260........
```

Fig. S15 Sequence alignment the members of GhSec137 orthologue groups. Sequence alignment was performed with using Clustal X2. Asterisks represent the absolutely conserved sites, and the dot (.) and (:) represents the variable sites with different or similar residue properties compared the consensus residue, respectively. The accession number of GhSec137

is D10G64907.
